# Iron therapy mitigates chronic kidney disease progression by regulating intracellular iron status of kidney macrophages

**DOI:** 10.1172/jci.insight.159235

**Published:** 2023-01-10

**Authors:** Edwin Patino, Divya Bhatia, Steven Z. Vance, Ada Antypiuk, Rie Uni, Chantalle Campbell, Carlo G. Castillo, Shahd Jaouni, Francesca Vinchi, Mary E. Choi, Oleh Akchurin

**Affiliations:** 1 Division of Nephrology and Hypertension, Joan and Sanford I. Weill Department of Medicine, Weill Cornell Medicine, New York, New York, USA.; 2 Iron Research Laboratory, Lindsley Kimball Research Institute, New York Blood Center, New York, New York, USA.; 3 Division of Pediatric Nephrology, Department of Pediatrics, Weill Cornell Medicine, New York, New York, USA.; 4 College of Agriculture and Life Sciences, Cornell University, Ithaca, New York, USA.; 5 Weill Cornell Medicine-Qatar, Cornell University, Doha, Qatar.; 6 Department of Pathology and Laboratory Medicine, Weill Cornell Medicine, New York, New York, USA.; 7 New York-Presbyterian Hospital, New York, New York, USA.

**Keywords:** Nephrology, Chronic kidney disease, Fibrosis, Macrophages

## Abstract

Systemic iron metabolism is disrupted in chronic kidney disease (CKD). However, little is known about local kidney iron homeostasis and its role in kidney fibrosis. Kidney-specific effects of iron therapy in CKD also remain elusive. Here, we elucidate the role of macrophage iron status in kidney fibrosis and demonstrate that it is a potential therapeutic target. In CKD, kidney macrophages exhibited depletion of labile iron pool (LIP) and induction of transferrin receptor 1, indicating intracellular iron deficiency. Low LIP in kidney macrophages was associated with their defective antioxidant response and proinflammatory polarization. Repletion of LIP in kidney macrophages through knockout of ferritin heavy chain (*Fth1*) reduced oxidative stress and mitigated fibrosis. Similar to *Fth1* knockout, iron dextran therapy, through replenishing macrophage LIP, reduced oxidative stress, decreased the production of proinflammatory cytokines, and alleviated kidney fibrosis. Interestingly, iron markedly decreased TGF-β expression and suppressed TGF-β–driven fibrotic response of macrophages. Iron dextran therapy and FtH suppression had an additive protective effect against fibrosis. Adoptive transfer of iron-loaded macrophages alleviated kidney fibrosis, validating the protective effect of iron-replete macrophages in CKD. Thus, targeting intracellular iron deficiency of kidney macrophages in CKD can serve as a therapeutic opportunity to mitigate disease progression.

## Graphical abstract

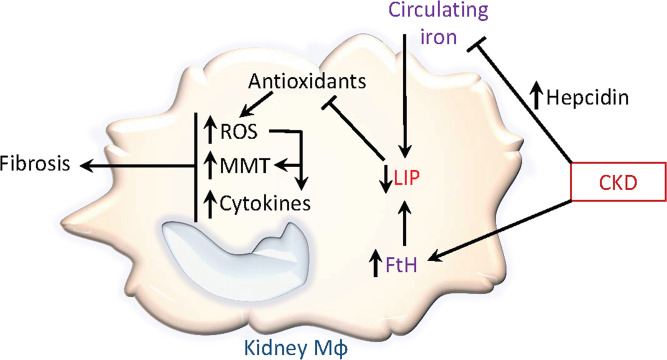

## Introduction

Chronic kidney disease (CKD) is a progressive multisystem disease that affects 10%–15% of the US adult population, has no cure, and is associated with high morbidity and mortality (1). Anemia and iron deficiency are common in patients with CKD (2, 3). Many patients with CKD develop functional iron deficiency, in part due to elevated hepcidin. Importantly, functional iron deficiency in CKD is independently associated with mortality (2). CKD-induced alterations of iron metabolism contribute to the development of anemia and require therapeutic correction (4). Iron administration becomes critical in patients receiving erythropoiesis-stimulating agents, which increase iron demand for de novo red blood cell production (4). At the same time, a growing body of literature indicates that iron has previously unrecognized erythropoiesis-independent effects in CKD (5), including those in CKD mineral and bone disorder, cardiovascular health, the immune system, and, importantly, kidney fibrosis (6, 7).

Recently, we reported that chronic parenteral administration of iron dextran improves kidney function in juvenile mice with adenine-induced CKD (8). Similarly, ferric citrate coordination complex improved kidney function in 5/6-nephrectomized rats (9) and in juvenile mice with Alport syndrome (10). Sucroferric oxyhydroxide ameliorated CKD progression in rats with adenine-induced CKD (11). However, despite this recent evidence suggesting a clinically relevant role for iron in the kidney, the cellular and molecular mechanisms responsible for potential iron-driven alleviation of kidney fibrosis have remained largely unexplored to date.

Kidney fibrosis is the final common pathway for all types of progressive kidney diseases. The deposition of excessive extracellular matrix during fibrosis destroys the normal architecture of kidney parenchyma, which results in a gradual loss of kidney function (12). Kidney macrophages participate in immune responses to chronic kidney injury (13). In response to stimuli from the microenvironment, macrophages shift their polarization and can adopt different functional phenotypes (14). In the setting of kidney fibrosis, macrophages may exhibit either profibrotic or protective effects (15). Subsets of kidney macrophages have been implicated in local inflammatory response, crosstalk with other immune cells, and the macrophage-to-myofibroblast transition (16). Moreover, kidney macrophages can become a potent source of oxidative stress that drives fibrosis, which is in part due to defective macrophage mitophagy in CKD (17).

Macrophages are also crucial for iron homeostasis (18). While the principal role of macrophages in supplying iron for erythropoiesis through systemic iron recycling is well established, growing evidence indicates that macrophages regulate local iron availability and thus modulate tissue microenvironment, affecting surrounding cells and tissue functions (19). Furthermore, iron may greatly impact macrophage polarization in several organs (20). Although iron therapy is being widely used in patients with CKD, the effects of iron on kidney macrophage behavior in the context of CKD remain largely unexplored (6). Owing to elevated hepcidin, macrophages may accumulate iron intracellularly in CKD (8). In macrophages, iron can join the labile iron pool (LIP), a cytosolic pool of free redox-active ferrous iron (Fe^2+^) (21). This dynamic pool of iron plays a regulatory role and is the source of iron for incorporation into iron-dependent enzymes, particularly many antioxidant enzymes (21). Upon its expansion, LIP is either exported by the iron exporter ferroportin or stored as nonreactive ferric iron (Fe^3+^) in ferritin nanocages (22). While elevated serum ferritin in CKD may reflect changes in iron status, ferritin levels are also affected by inflammation (23). In this study, using 2 mouse models of CKD, a genetic manipulation of myeloid lineage–specific ferritin heavy chain and a complementary cell culture system, we examined the impact of CKD on kidney macrophage intracellular iron status and its effect on macrophage behavior and progression of kidney fibrosis. Furthermore, we elucidated cellular effects of therapeutic iron administration on kidney macrophage-elicited inflammatory and fibrotic responses in CKD.

## Results

### The intracellular LIP is depleted in kidney macrophages during CKD.

**Figure 1:**
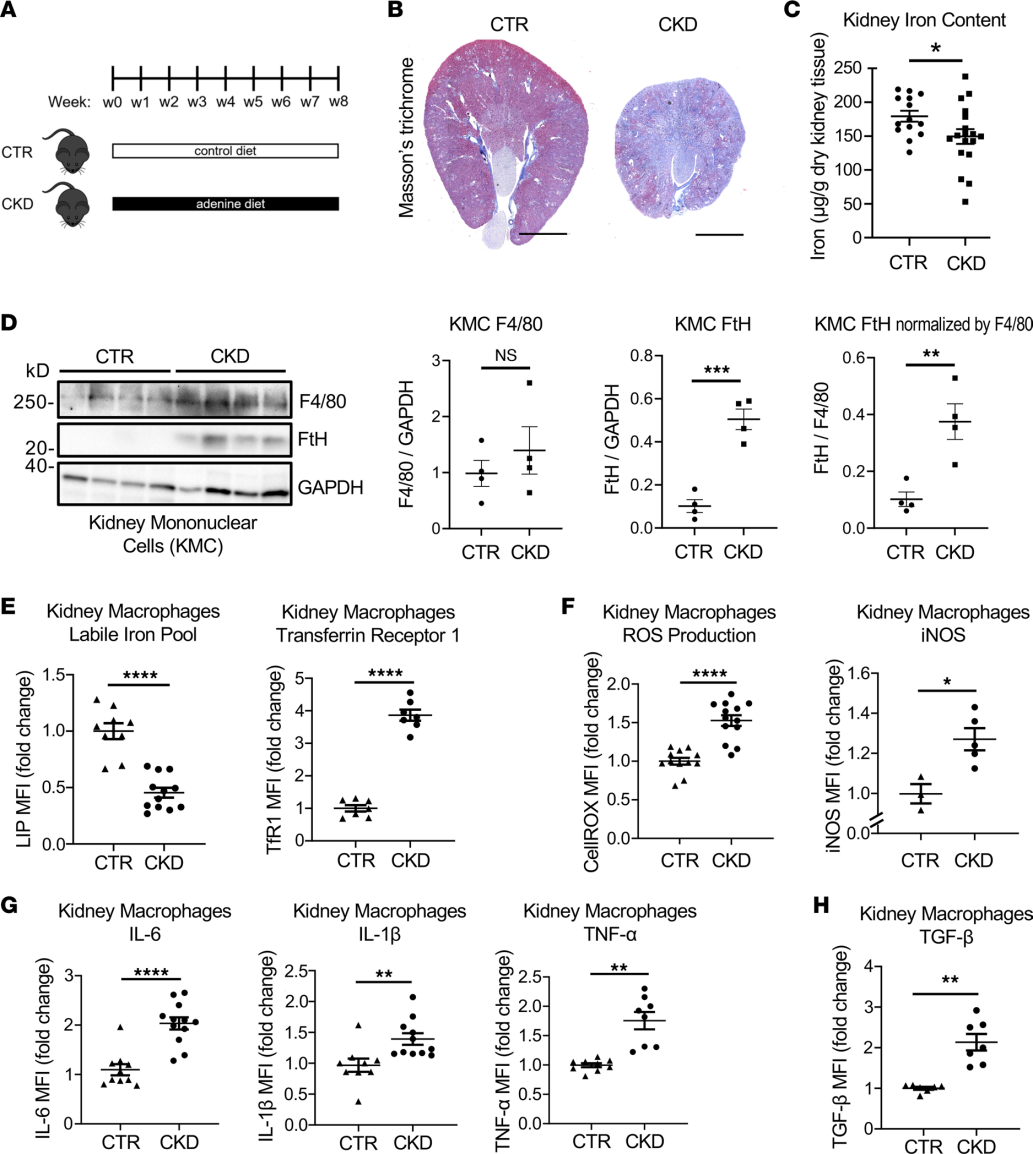
In mice with CKD, kidney macrophages display depletion of LIP associated with oxidative stress and inflammation. (**A**) Schematic diagram of CKD induction by the 0.2% adenine diet. (**B**) Trichrome staining of control (CTR) and CKD kidneys. Scale bars, 2.5 mm. (**C**) Kidney iron content in 2 groups of mice at euthanasia. (**D**) Expression of ferritin heavy chain (FtH) protein in isolated kidney mononuclear cells (KMC, macrophages/monocytes, and lymphocytes) normalized by macrophage marker F4/80 and by GAPDH. KMCs were isolated using Ficoll density gradient centrifugation of kidney single-cell suspensions. (**E**) Kidney macrophage labile iron pool (LIP) and transferrin receptor 1 (TfR1 or CD71) expression in CTR and CKD groups. (**F**) ROS production and iNOS expression in kidney macrophages in CTR and CKD groups. (**G**) Production of proinflammatory cytokines IL-6, IL-1β, and TNF-α in kidney macrophages, CTR and CKD groups. (**H**) TGF-β expression in kidney macrophages in CTR and CKD groups. Error bars represent SEM. Data were analyzed using *t* test. *P < 0.05; **P < 0.01; ***P < 0.001; ****P < 0.0001. MFI, mean fluorescence intensity.

We used a high-adenine diet mouse model of CKD (8) (Figure 1A). CKD mice developed severe kidney fibrosis (Figure 1B) and displayed systemic hypoferremia with low serum iron, low transferrin saturation, and high serum soluble transferrin receptor levels, (Supplemental Figure 1; supplemental material available online with this article; https://doi.org/10.1172/jci.insight.159235DS1). The number of macrophages in the kidney was significantly increased in CKD as compared with control mice (Supplemental Figure 2). Due to the critical role of macrophages in both kidney fibrosis and iron homeostasis, we analyzed the intracellular iron status as well as oxidative stress and inflammation in kidney macrophages during CKD. Kidney tissue iron content was decreased in CKD (Figure 1C) while ferritin heavy chain (FtH) expression was induced by CKD in kidney mononuclear cells (Figure 1D). Consistent with decreased kidney iron content, kidney macrophages in CKD showed intracellular iron deficiency as indicated by lower LIP and higher transferrin receptor 1 (TfR1 or CD71) compared with control kidney macrophages (Figure 1E). This was associated with increased intracellular oxidative stress, indicated by elevated reactive oxygen species (ROS) and inducible nitric oxide synthase (iNOS) levels (Figure 1F), as well as induction of the proinflammatory cytokines IL-6, IL-1β, and TNF-α (Figure 1G) and TGF-β (Figure 1H) in kidney macrophages of CKD mice compared with controls. Interestingly, these CKD-induced phenotypic changes were not unique to kidney macrophages, as suggested by a similar pattern of LIP depletion and proinflammatory polarization of spleen macrophages in CKD compared with control mice (Supplemental Figure 3). Overall, these results indicate that CKD induces intracellular iron deficiency in macrophages associated with their proinflammatory and profibrotic activation.

### Myeloid Fth1 deletion replenishes LIP in CKD kidney macrophages and attenuates kidney fibrosis.

**Figure 2:**
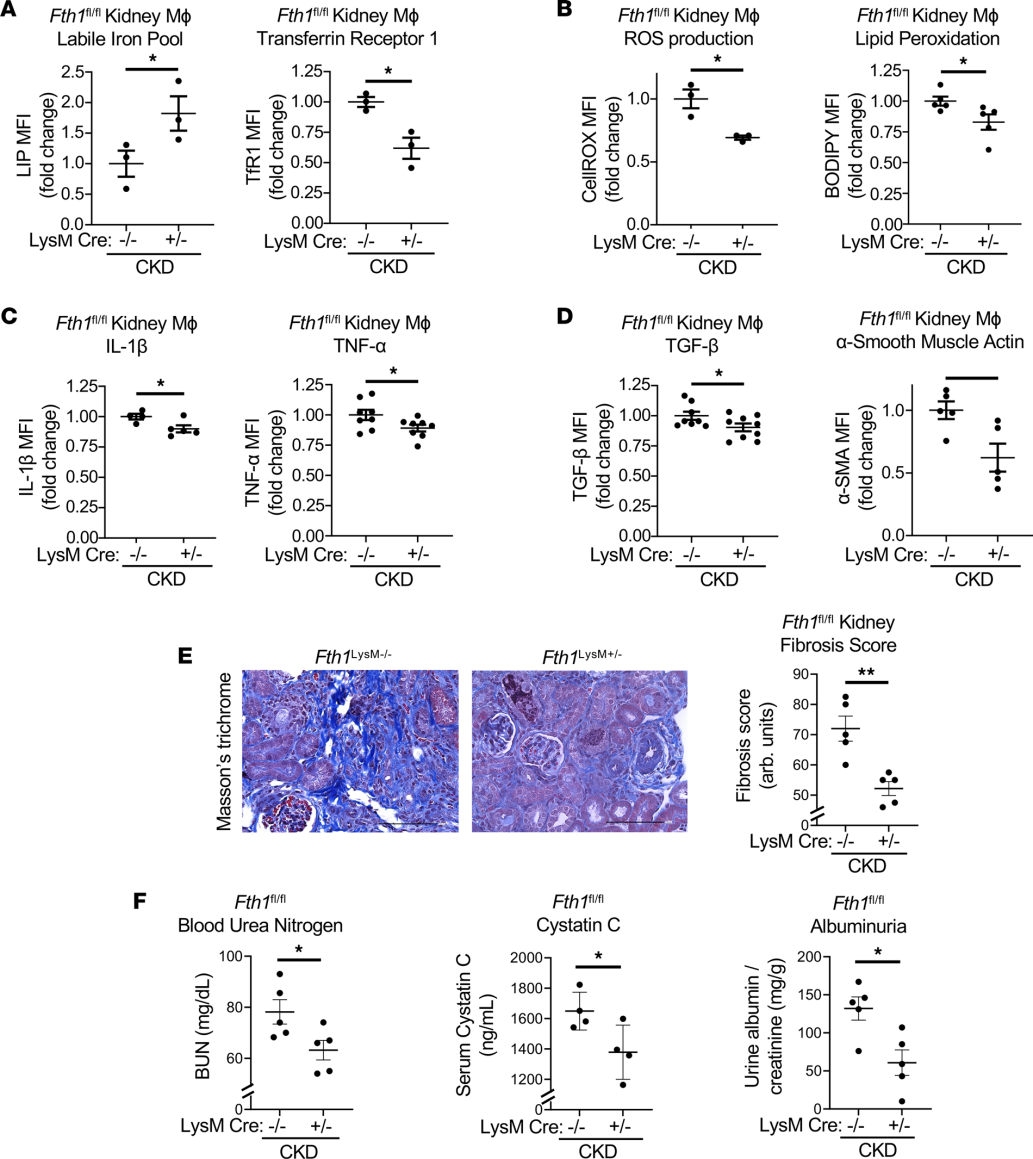
Myeloid-specific deletion of *Fth1* improves iron deficiency of kidney macrophages and mitigates kidney fibrosis. (**A**) Myeloid *Fth1* deletion replenished the intracellular LIP and reduced TfR1 expression by kidney macrophages; *n* = 3 per group. One-tailed *t* test. *P < 0.05. (**B**) ROS and lipid peroxidation in kidney macrophages of CKD *Fth1*^LysM–/–^ and *Fth1*^LysM+/–^ CKD mice; *n* = 3–5 per group. (**C**) Expression of IL-1β and TNF-α by kidney macrophages in 2 groups of mice; *n* = 4–8 per group. (**D**) Fibrosis markers TGF-β and α–smooth muscle actin (α-SMA) in kidney macrophages of CKD *Fth1*^LysM–/–^ and *Fth1*^LysM+/–^ CKD mice; *n* = 5–9 per group. (**E**) Representative images of Masson’s trichrome staining of kidney sections in 2 groups of mice; the quantification is shown as kidney fibrosis score; *n* = 5 per group. (**F**) Biomarkers of the kidney function: urine albumin to creatinine ratio (ACR), blood urea nitrogen (BUN), and serum cystatin C in CKD *Fth1*^LysM–/–^ and *Fth1*^LysM+/–^ mice; *n* = 5 per group. ACR and cystatin C were measured after 4 weeks of adenine diet, BUN after 8 weeks of adenine diet. Scale bars, 100 μm. Error bars represent SEM. Data were analyzed using *t* test. *P < 0.05; **P < 0.01.

To better understand the pathogenic role of an altered kidney macrophage LIP versus ferritin-stored iron in kidney fibrosis, we next examined the effect of FtH suppression in macrophages by inducing CKD in myeloid specific *Fth1*-knockout mice (*Fth1*^LysM+/–^) (Figure 2 and Supplemental Figures 4–6). FtH deletion was confirmed by Western blot in cultured bone marrow–derived macrophages (BMDMs) from *Fth1*^LysM+/–^ mice compared with *Fth1*^LysM–/–^ (Supplemental Figure 4B). As expected, accumulation of ferric iron in the spleen of *Fth1*^LysM–/–^ CKD mice was reduced in *Fth1*^LysM+/–^ CKD mice (Supplemental Figure 4C) and associated with improvement of anemia (Supplemental Figure 4D). The lack of FtH ferroxidase activity, which converts reactive ferrous Fe^2+^ into inert ferric Fe^3+^, is expected to increase intracellular LIP by inhibiting iron deposition in ferritin nanocages. Indeed, myeloid *Fth1* deletion rescued intracellular iron deficiency in CKD kidney macrophages as it improved LIP and reduced TfR1 (Figure 2A). This was associated with reduced oxidative stress assessed by ROS production and lipid peroxidation (Figure 2B) and decreased production of inflammatory cytokines and TGF-β (Figure 2, C and D). *Fth1* deletion also reduced the expression of α–smooth muscle actin (α-SMA) in kidney macrophages in CKD (Figure 2D and Supplemental Figure 5), suggesting that myeloid *Fth1* deletion suppressed kidney macrophage-to-myofibroblast transition in CKD. Consequently, kidney fibrosis and kidney function, assessed by trichrome staining, urine albumin-to-creatinine ratio (ACR), blood urea nitrogen (BUN), and serum cystatin C, were improved in *Fth1*^LysM+/–^ CKD mice compared with *Fth1*^LysM–/–^ CKD mice (Figure 2, E and F). Similar differences were observed in *Fth1*^LysM+/–^ spleen macrophages compared with *Fth1*^LysM–/–^ ones in CKD mice (Supplemental Figure 6). Thus, myeloid *Fth1* deletion replenished LIP in macrophages and attenuated kidney fibrosis. These results indicate that the maintenance of LIP levels but not the stored iron in macrophages is critical for the control of macrophage-elicited inflammatory and fibrotic responses in CKD.

### Iron dextran therapy replenishes LIP in kidney macrophages and reduces their profibrotic responses in CKD.

**Figure 3:**
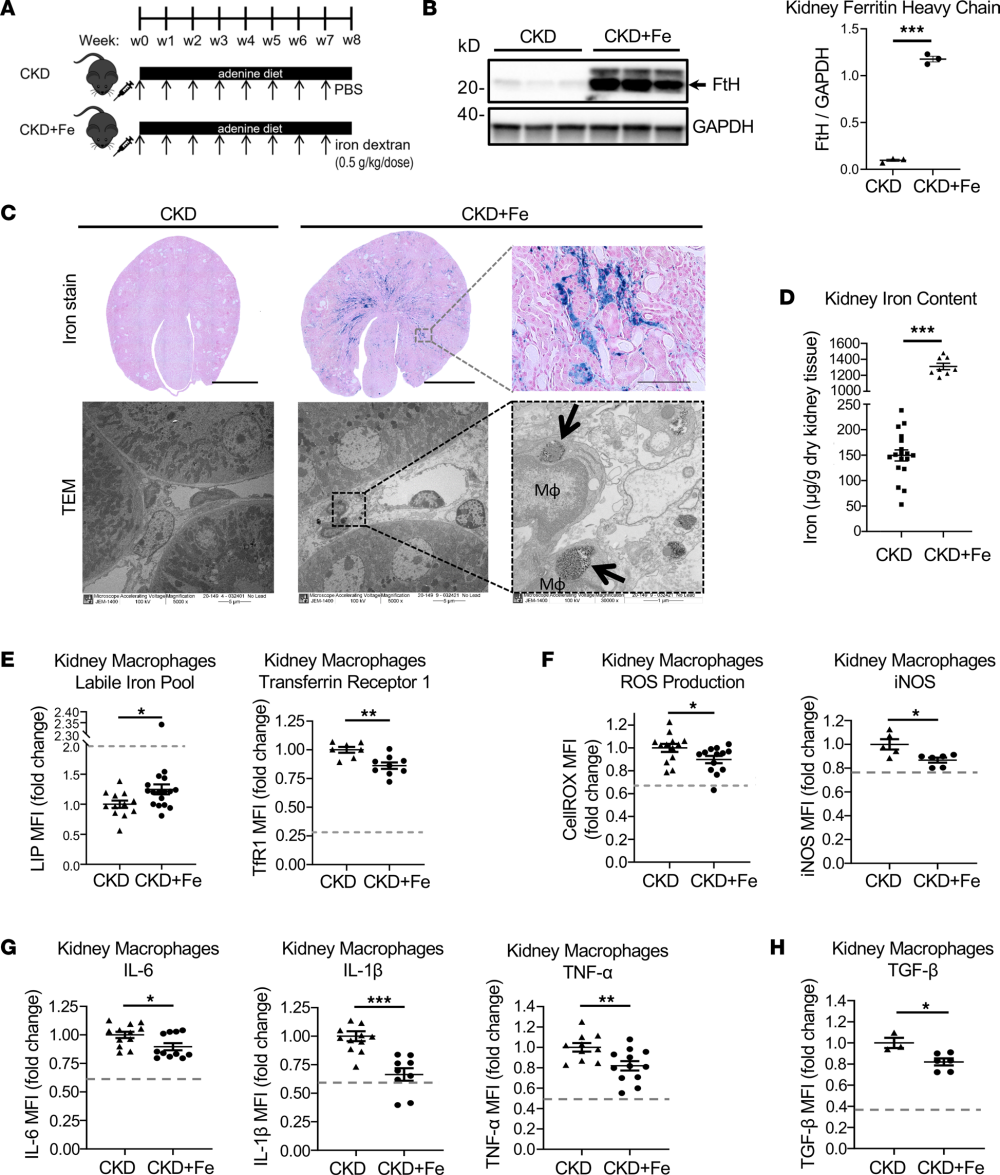
Iron dextran administration improves iron deficiency of kidney macrophages and reduces kidney macrophage oxidative stress and inflammation in mice with CKD. (**A**) Schematic diagram of iron dextran administration in mice with adenine-induced CKD and (**B**) Western blot analysis of FtH protein levels in kidney tissue of CKD mice in the absence (CKD) and presence (CKD+Fe) of iron administration; *n* = 3 per group. (**C**) Perls Prussian blue staining for ferric iron (scale bars: 3 mm for low magnification, 100 μm for high magnification) and transmission electron microscopy (original magnification, 5,000×, first 2 images, and 30,000×, last image) of kidney tissue of CKD mice in the absence and presence of iron administration. Arrows point at iron-loaded lysosomes within kidney macrophages (Mϕ). (**D**) Kidney tissue iron content in 2 groups of mice (*n* = 8–18 per group). (**E**) Iron therapy increases LIP and decreases TfR1 expression in CKD kidney macrophages, thus improving their iron deficiency status; *n* = 7–16 per group. (**F**) ROS and iNOS expression in CKD kidney macrophages in the absence and presence of iron therapy; *n* = 5–13 per group. (**G**) Iron therapy inhibits production of proinflammatory cytokines IL-6, IL-1β, and TNF-α in CKD kidney macrophages. (**H**) TGF-β expression in CKD kidney macrophages is suppressed by iron therapy. Dashed gray lines indicate mean values of the respective parameters in control mice (**E**–**H**). Error bars represent SEM. Data were analyzed using *t* test. *P < 0.05; **P < 0.01; ***P < 0.001.

We then tested whether therapeutic iron administration in CKD achieved beneficial effects similar to myeloid *Fth1* deletion through correction of macrophage iron status. Iron dextran was administered intraperitoneally (i.p.) weekly at the previously tested dose (0.5 g/kg) that was adequate for the correction of anemia in mice with adenine-induced CKD (8) (Figure 3A). As expected, iron therapy further increased kidney FtH expression in CKD mice (Figure 3B). Increased tissue FtH expression reflected iron accumulation in kidney tissue as revealed by histology (Figure 3C) and by the measurement of kidney tissue iron content (Figure 3D). In the tubulointerstitial compartment, iron deposition was prominent in peritubular areas. Specifically, iron accumulated in kidney macrophages, based on transmission electron microscopy (Figure 3C). Iron dextran uptake by macrophages in mice with CKD improved intracellular iron status, as indicated by expanded LIP and decreased expression of TfR1 (Figure 3E). This was associated with reduced oxidative stress, assessed by ROS and iNOS levels (Figure 3F), and reduced production of the proinflammatory cytokines IL-6, IL-1β, and TNF-α (Figure 3G), as well as TGF-β (Figure 3H). These results indicate that iron therapy, by promoting iron uptake by kidney macrophages and replenishing the intracellular LIP, corrected macrophage iron deficiency. Consistent with the effects of myeloid specific *Fth1* deletion, replenishment of LIP by iron therapy was accompanied by an attenuated proinflammatory and profibrotic activation of kidney macrophages.

### Correction of macrophage iron status by iron dextran therapy is associated with slowing CKD progression.

**Figure 4:**
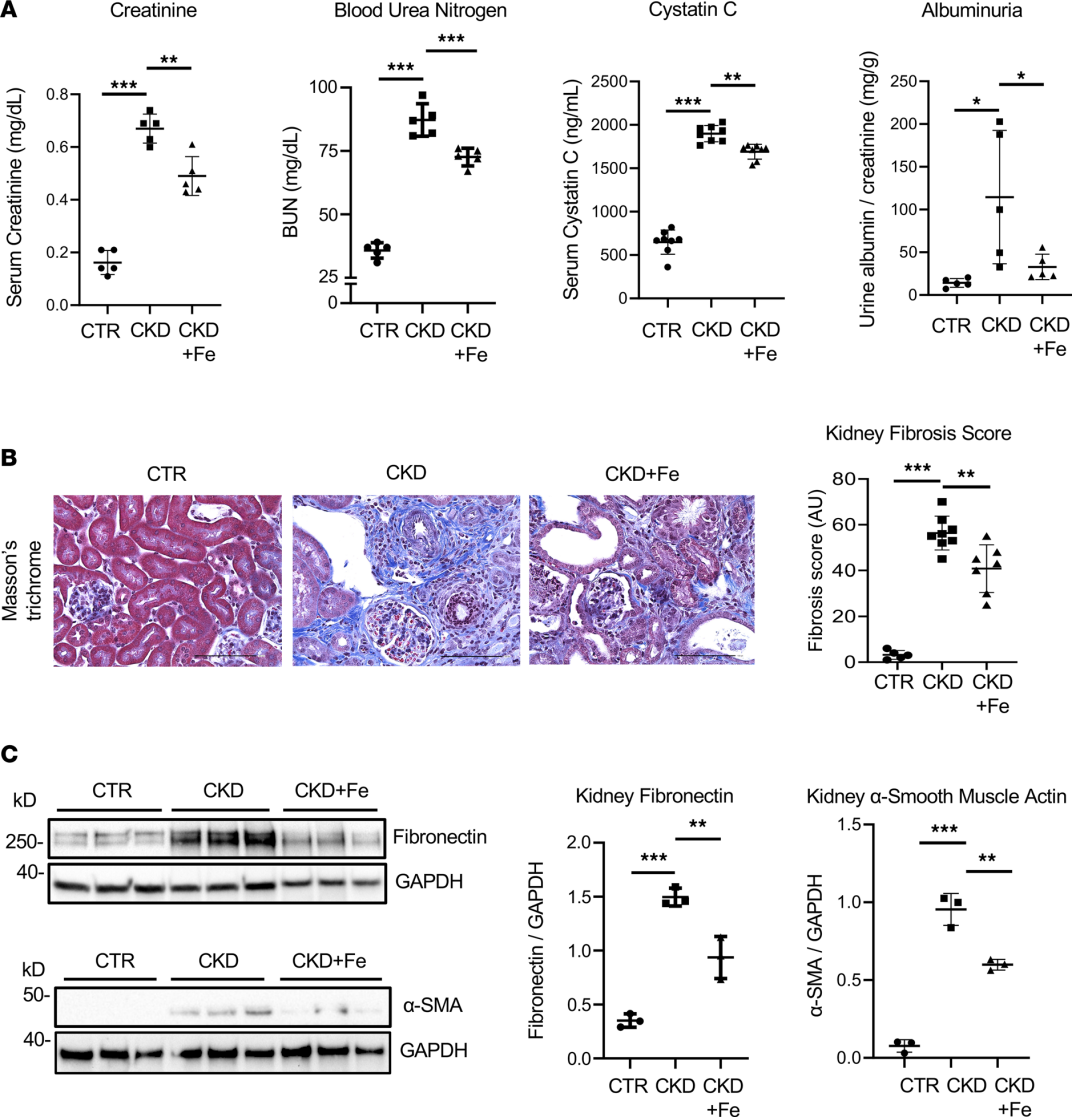
Iron dextran therapy improves kidney fibrosis and kidney function in mice with CKD. (**A**) Markers of kidney function: serum creatinine, BUN, serum cystatin C, and urine ACR in CTR mice and mice with CKD in the absence and presence of iron administration; *n* = 5–8 per group. (**B**) Representative images of Masson’s trichrome staining of kidney sections of CTR mice and mice with CKD in the absence and presence of iron administration; the quantification of blue-stained collagen is shown as kidney fibrosis score; *n* = 5–8 per group. Scale bars, 100 μm. (**C**) Western blot analysis of fibronectin and α–smooth muscle actin in kidney tissue of CTR mice and mice with CKD in the absence and presence of iron administration; *n* = 3 per group. Iron dextran was administered i.p. once a week, 0.5 g/kg (CKD Fe group). Error bars represent SD. Data were analyzed using ANOVA; *P < 0.05; **P < 0.01; ***P < 0.001.

To examine the effects of iron therapy on CKD progression, we analyzed kidney function, histology, and expression of extracellular matrix proteins in kidney tissue in the presence or absence of iron therapy. Iron therapy improved kidney function, assessed by serum creatinine, BUN, serum cystatin C, and urine ACR (Figure 4A) and attenuated kidney fibrosis in mice with CKD, as indicated by reduced collagen deposition (Figure 4B) and reduced expression of kidney fibronectin and α-SMA in iron-treated compared with untreated CKD mice (Figure 4C). Iron therapy reduced α-SMA expression not only in whole kidney tissue but also specifically in kidney mononuclear cells (Supplemental Figure 7A) and appeared to decrease coexpression of α-SMA and F4/80 in kidney tissue (Supplemental Figure 7B), suggesting that iron dextran administration reduced kidney macrophage-to-myofibroblast transition in CKD. Treatment with iron dextran also improved body weight (Supplemental Figure 8) and anemia (Supplemental Figure 9) in mice with CKD, without altering the total leukocyte count or percentages of circulating monocytes, lymphocytes, and neutrophils (Supplemental Table 1). These data demonstrate disease-modifying effects of iron homeostasis in CKD, as iron deficiency aggravated and iron dextran administration alleviated kidney fibrosis and improved kidney function.

Since both the myeloid specific FtH deficiency and the iron therapy improved kidney fibrosis, we asked whether additional suppression of macrophage FtH in the presence of iron therapy would further attenuate kidney fibrosis. To test this, we subjected *Fth1*^LysM+/–^ and *Fth1*^LysM–/–^ CKD mice to iron therapy (Supplemental Figure 10A). Hemoglobin remained comparable in the 2 groups (Supplemental Figure 10B). Myeloid *Fth1* deletion combined with iron therapy in CKD mice resulted in further improvement of kidney function as assessed by BUN (Supplemental Figure 10C) and further reduction of kidney fibrosis as evaluated by Masson’s trichrome staining (Supplemental Figure 10D) compared with iron therapy alone, indicating a synergistic antifibrotic effect of myeloid *Fth1* deletion and iron therapy. These data also suggest that effects of iron therapy in kidney macrophages and consecutive improvement of fibrosis were not mediated by the ferritin-bound fraction of iron in macrophages.

### Correction of macrophage iron status improves kidney fibrosis in the unilateral ureteral obstruction model.

**Figure 5:**
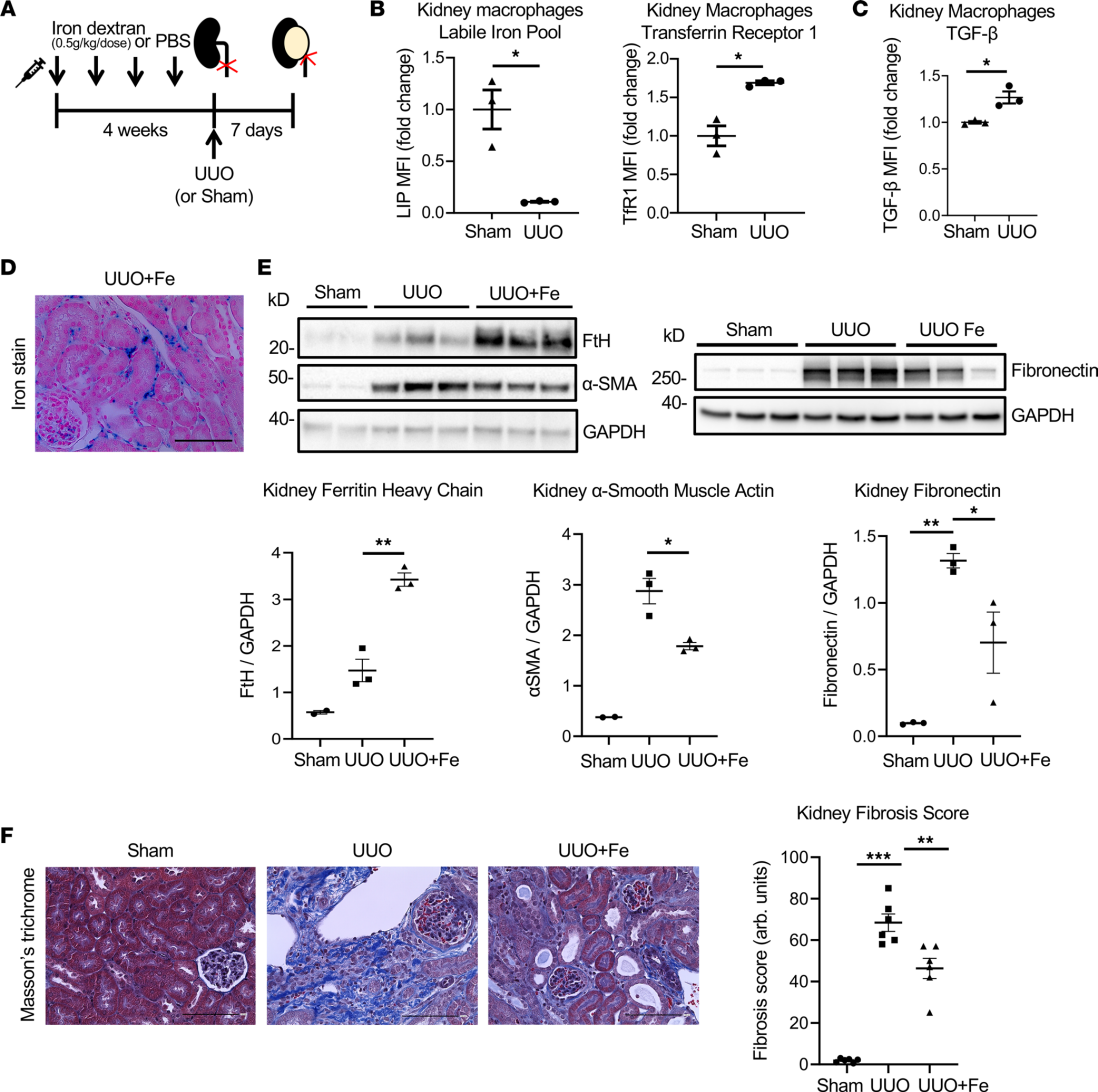
Effects of iron administration on kidney fibrosis in the UUO model. (**A**) Mice were pretreated with iron dextran (0.5 g/kg/dose weekly) or PBS for 4 weeks prior to surgery. Kidneys were harvested 7 days after the UUO or sham surgeries. (**B**) LIP, TfR1, and (**C**) TGF-β expression in kidney macrophages (*n* = 3 per group). (**D**) Perls Prussian blue staining demonstrates ferric iron accumulation in the interstitial spaces in the obstructed kidneys of UUO+Fe mice. Scale bar, 100 μm. (**E**) Immunoblotting of kidney tissues for FtH, fibronectin, and α-SMA protein expression in the obstructed kidneys of UUO mice that received iron dextran injections (UUO+Fe) compared with 2 other groups of mice. (**F**) Histologic assessment of kidney fibrosis in 3 groups of mice with Masson’s trichrome staining; quantification (*n* = 6 per group) and representative images. Scale bars, 100 μm. Data were analyzed using *t* test (**B** and **C**) or ANOVA (**E** and **F**). Error bars represent SEM; *P < 0.05; **P < 0.01; ***P < 0.001.

To further validate our findings, we used an additional mouse model of kidney fibrosis, the unilateral ureteral obstruction (UUO) model (Figure 5A and Supplemental Figure 11, A and B). Similar to the adenine model, kidney macrophages showed intracellular iron depletion with low LIP and elevated TfR1 in the UUO model (Figure 5B). This depletion of kidney macrophage LIP was associated with proinflammatory and profibrotic activation, characterized by induction of IL-6, IL-1β, and TGF-β (Figure 5C and Supplemental Figure 11C). Upon iron dextran administration, the pattern of iron accumulation in obstructed kidneys (Figure 5, D and E) was similar to that observed in the adenine model. Myeloid *Fth1* deletion corrected the iron status in UUO kidney macrophages and reduced their proinflammatory and profibrotic activation, as indicated by lower IL-6, IL-1β, and TGF-β levels (Supplemental Figure 12), similar to the adenine model. Thus, replenishment of macrophage LIP achieved by iron dextran administration in UUO mice had a therapeutic antifibrotic effect in the kidney, as indicated by the improved fibrosis, assessed by expression of fibronectin and α-SMA in kidney tissue (Figure 5E) and by histology (Figure 5F).

### Iron dextran therapy improves renal antioxidant response in CKD.

**Figure 6:**
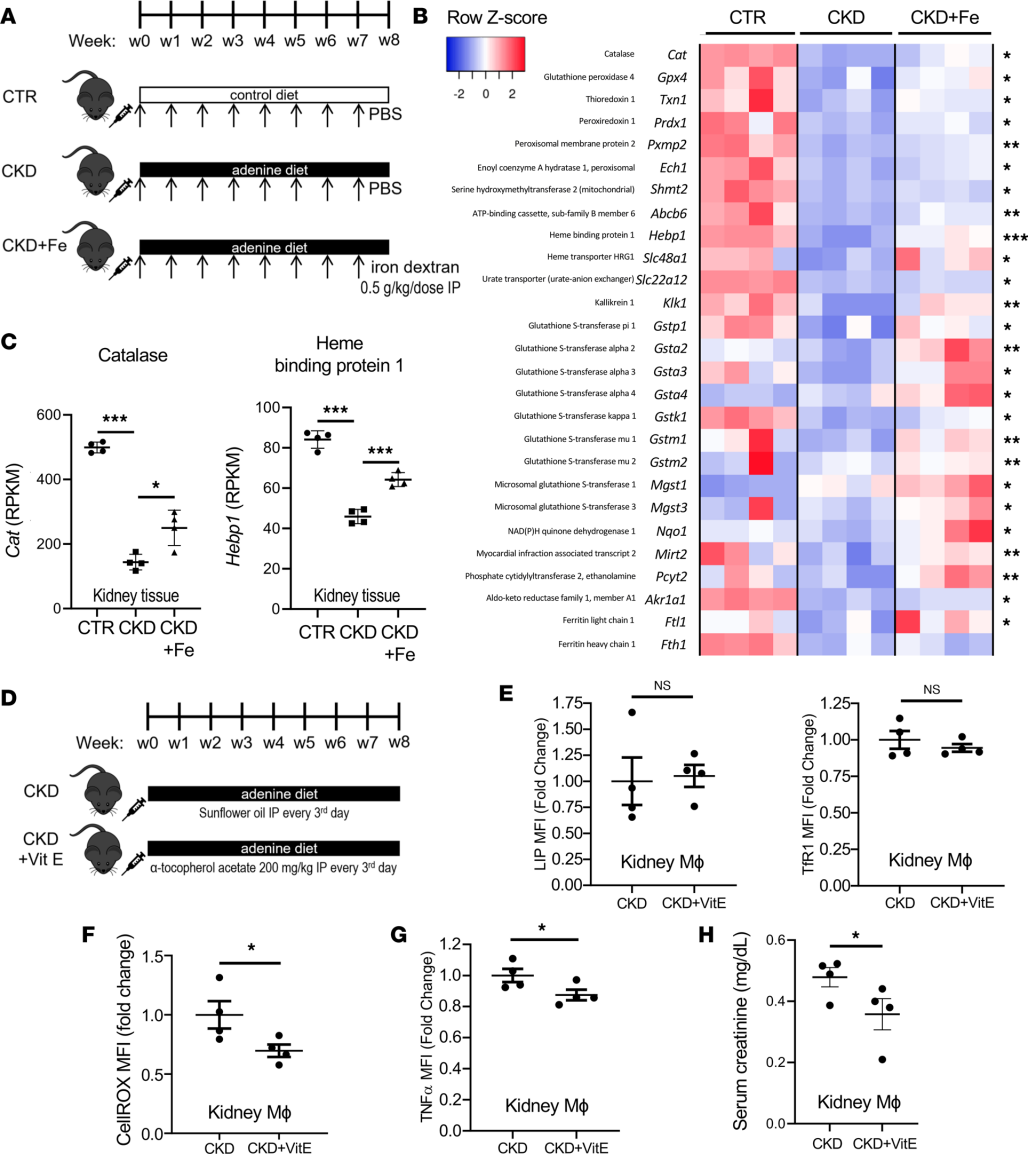
Iron administration and renal antioxidant response in mice with CKD. (**A**) CKD was induced by a 0.2% adenine diet, and kidney tissues were processed for RNA sequencing. (**B**) Heatmap showing expression of top differentially expressed antioxidant genes in the kidneys of CTR mice, untreated CKD mice, and CKD mice that received weekly i.p. injections of iron dextran (CKD+Fe). Blue color represents low expression and red color high expression; *P < 0.05; **P < 0.01; ***P < 0.001 for differences between CKD and CKD+Fe groups. (**C**) Expression of selected genes catalase and heme binding protein 1; *n* = 4 per group. (**D**) Schematic diagram of α-tocopherol (vitamin E), an antioxidant, administration in CKD mice. (**E**) α-Tocopherol administration did not change LIP and TfR1 expression in kidney macrophages but (**F**) reduced their ROS and (**G**) TNF-α expression and (**H**) improved serum creatinine. Error bars represent SEM. *P < 0.05. RPKM, reads per kilobase of transcript per million mapped reads.

Because we observed enhanced ROS production by kidney macrophages in CKD and their reduction by iron therapy, we evaluated ROS implication in the proinflammatory polarization of iron-deficient macrophages. To this end, we first examined effects of CKD on kidney antioxidant response and the impact of iron therapy (Figure 6A). Kidney tissue RNA-sequencing data revealed that while many antioxidant genes were severely downregulated in kidney tissue of CKD mice, the expression of these genes increased in the kidney after iron dextran therapy (Figure 6B). Specifically, iron therapy induced the transcription of peroxisomal antioxidant enzymes including catalase, GSTK1, and MGST1; ferroptosis inhibitors GPX4 and MGST1; heme transporter SLC48a1 and heme binder HEBP1; as well as enzymes involved in glutathione reduction, such as GSTA2, GSTM1, and GSTM2 (Figure 6, B and C). These findings indicate that the beneficial effect of iron dextran therapy in CKD is likely mediated by its ability to counteract the toxic effect of CKD-driven ROS through the induction of kidney antioxidant response, which is severely suppressed in this condition.

To further evaluate the involvement of ROS in macrophage activation in the adenine model of CKD, we subjected CKD mice to the administration of vitamin E (α-tocopherol), an antioxidant (Figure 6D). While vitamin E did not change systemic (Supplemental Figure 13B) or kidney macrophage-specific iron status (Figure 6E), its ability to reduce CKD-induced ROS production (Figure 6F) was associated with reduced inflammatory cytokine levels in kidney macrophages (Figure 6G, Supplemental Figure 13C) and improved kidney function (Figure 6H), in a manner comparable to that obtained through iron therapy (Figure 3). Overall, these data suggest that macrophage iron deficiency induces inflammatory and fibrotic cell activation through inappropriate antioxidant response and ROS elevation; the latter can be corrected by iron dextran or antioxidant administration.

### Iron inhibits the profibrotic response of macrophages to TGF-β in vitro.

**Figure 7:**
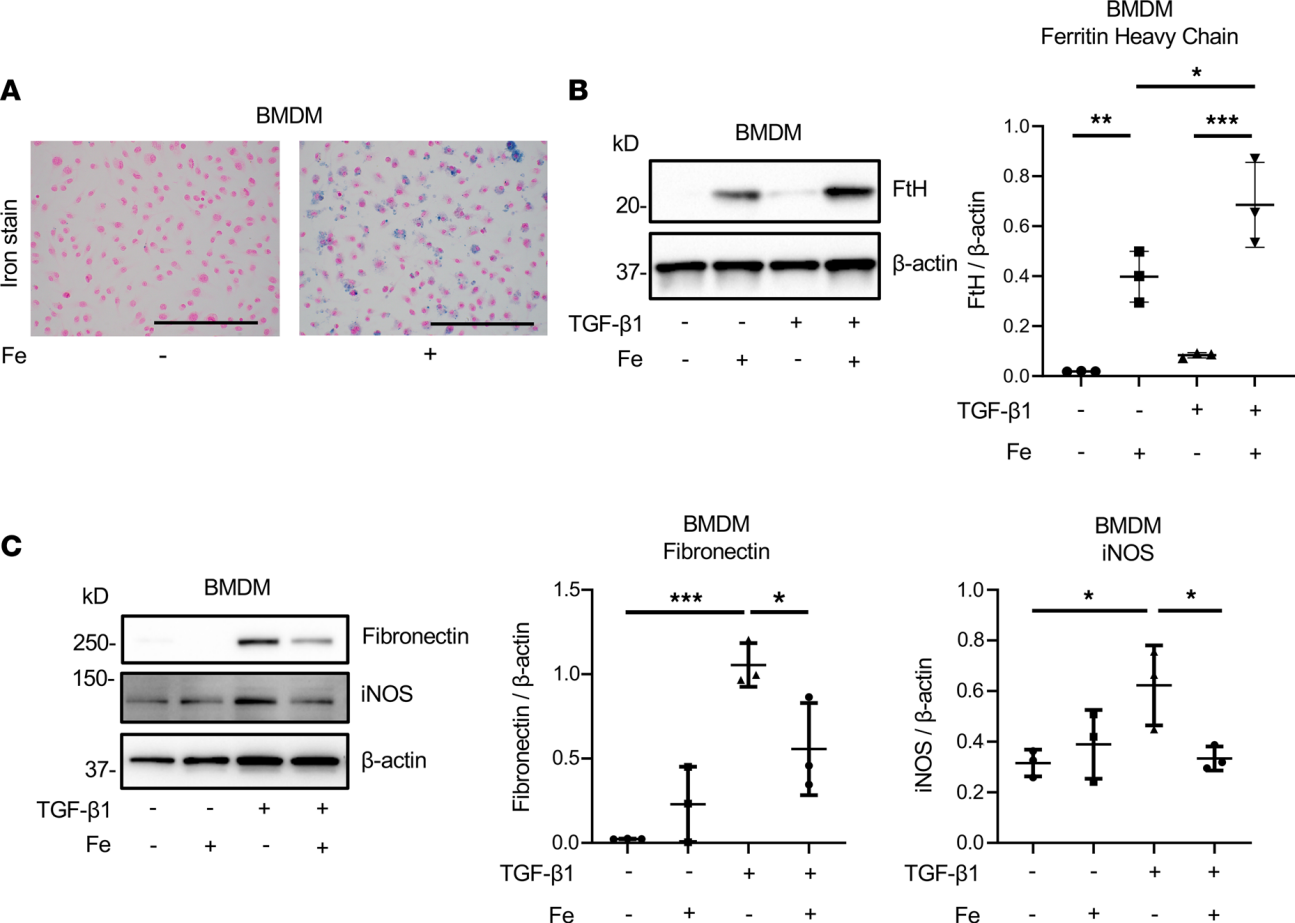
Iron attenuates profibrotic response to TGF-β in macrophages. BMDMs were left untreated (Fe-) or exposed to 25 mM ferric ammonium citrate (Fe+) for 20 hours. (**A**) Perls Prussian blue staining for ferric iron in control and iron-loaded BMDMs. Scale bars, 100 μm. (**B**) Western blot analysis of FtH, (**C**) fibronectin, and iNOS proteins in control BMDMs and BMDMs exposed to iron and TGF-β1 alone (5 ng/mL) or combined. Statistical analysis and representative blots. Immunoblotting was repeated 3 times. Error bars represent SD. *P < 0.05; **P < 0.01; ***P < 0.001.

Our in vivo data indicated that iron therapy mitigated kidney fibrosis. While this effect can be attributed to iron-mediated reduction of TGF-β production by kidney macrophages, we sought to examine whether iron also affects the profibrotic response of macrophages to TGF-β. To test this, we exposed murine BMDMs to iron for 20 hours in the presence or absence of TGF-β1 stimulation (5 ng/mL). BMDMs exposed to iron displayed intracellular iron accumulation, as demonstrated by Perls staining (Figure 7A) and FtH induction, the latter more pronounced in the presence of TGF-β1 stimulation (Figure 7B). Interestingly, TGF-β1 alone induced FtH in BMDMs but reduced their LIP (Supplemental Figure 14), suggesting that FtH activation contributes to macrophage iron depletion in CKD. While TGF-β1 alone induced fibronectin expression in BMDMs, coexposure with iron significantly attenuated fibronectin induction (Figure 7C). iNOS expression was induced by TGF-β1 and reduced to basal level by cotreatment with iron (Figure 7C). These data indicate that iron exposure alleviates the profibrotic response of macrophages to TGF-β.

### Adoptive transfer of iron-loaded macrophages mitigates kidney fibrosis.

**Figure 8:**
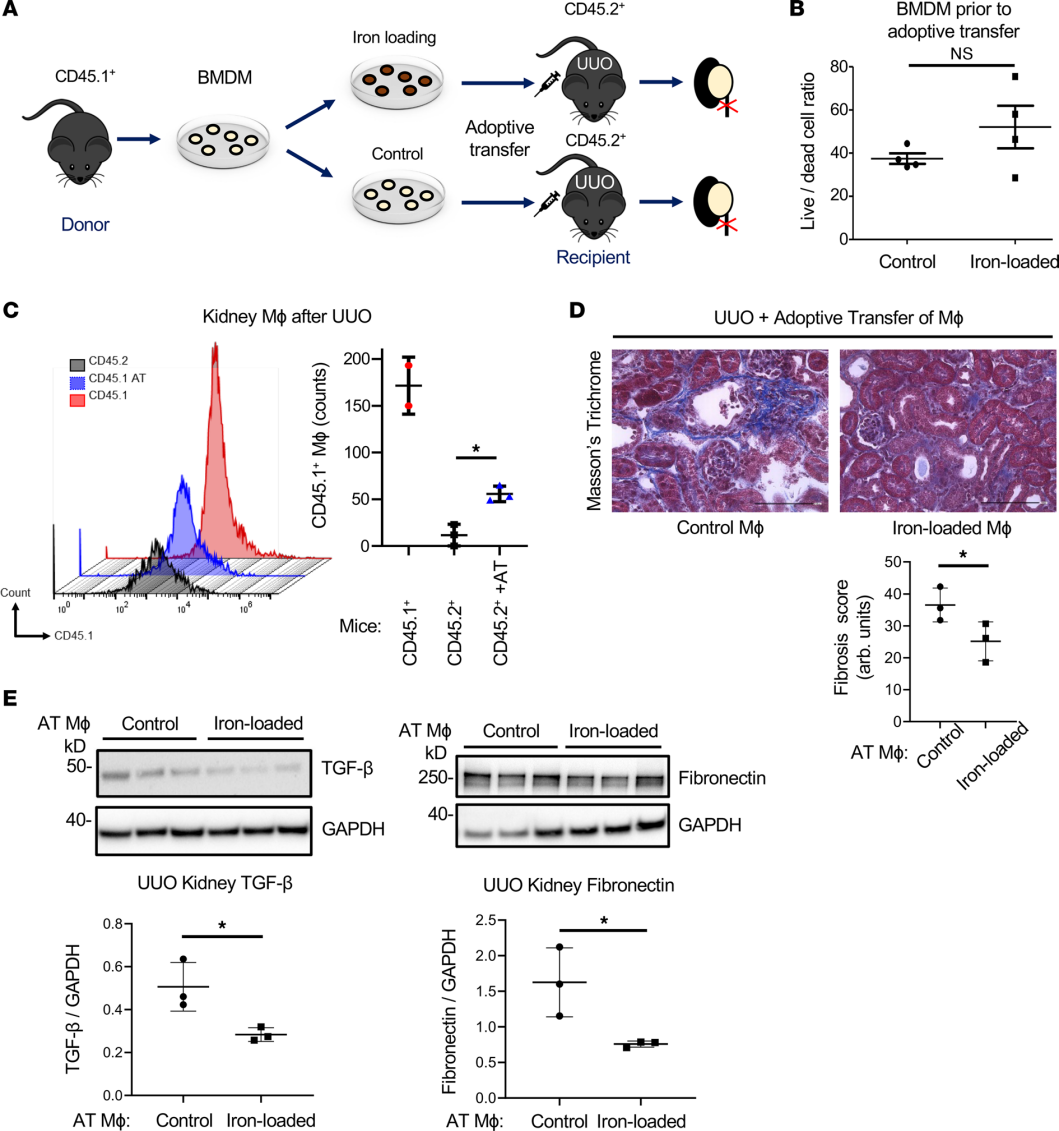
Adoptive transfer of iron-loaded macrophages limits kidney fibrosis in mice subjected to UUO. (**A**) Schematic diagram of the adoptive transfer (AT) of iron-loaded macrophages into UUO mice. BMDMs were isolated from CD45.1^+^ mice and left untreated (control) or treated with 25 μM ferric ammonium citrate (iron-loaded) for 20 hours prior to AT. Kidney fibrosis was induced by UUO in CD45.2^+^ mice. CD45.2^+^ mice received intravenously control or iron-loaded CD45.1^+^ macrophages. Kidneys were harvested 5 days after AT. (**B**) The ratio between live and dead cells in control versus iron-loaded BMDMs prior to transfer was assessed by flow cytometry using DAPI; *n* = 4 per group. (**C**) Flow cytometry histogram and count of CD45.1^+^CD11b^+^F4/80^+^ macrophages in recipient CD45.2^+^ kidneys upon AT of control macrophages. (**D**) Assessment of UUO-induced kidney fibrosis by Masson’s trichrome staining after AT of control versus iron-loaded macrophages. (**E**) Expression of latent TGF-β and fibronectin in kidney tissues after AT of control versus iron-loaded macrophages. Scale bars, 100 μm. Error bars represent SD; *n* = 3 per group. Data were analyzed using *t* test. *P < 0.05.

Our findings indicate that iron loading attenuates the proinflammatory and profibrotic properties of kidney macrophages in CKD (Figure 7). To further evaluate the therapeutic potential of iron loading in macrophages against kidney fibrosis, we performed adoptive transfer of iron-loaded macrophages in mice subjected to UUO (Figure 8A and Supplemental Figure 15A). CD45.1^+^ BMDMs were pretreated with 25 μM ferric ammonium citrate for 20 hours. No reduction of cell viability after iron exposure was observed (Figure 8B). CD45.2^+^ recipient mice received either control or iron-loaded CD45.1^+^ BMDMs 1 hour after UUO by intravenous (i.v.) infusion. Flow cytometry analysis of kidney single-cell suspensions from UUO kidneys demonstrated infiltration of kidney tissues by CD45.1^+^ macrophages following adoptive transfer (Figure 8C). This was confirmed by immunofluorescence staining of CD45.1^+^ F4/80^+^ macrophages in kidney tissues (Supplemental Figure 15B). Kidney fibrosis was attenuated in UUO kidneys of mice receiving iron-loaded compared with control macrophages, as indicated by reduced collagen deposition (Masson’s trichrome staining, Figure 8D). Accordingly, kidney expression of TGF-β and fibronectin (Figure 8E) was reduced by adoptive transfer of iron-loaded compared with control macrophages. These results highlight the protective effect of iron-loaded macrophages against kidney fibrosis in CKD and the therapeutic potential of targeting macrophage iron depletion to alleviate CKD progression.

## Discussion

**Figure 9:**
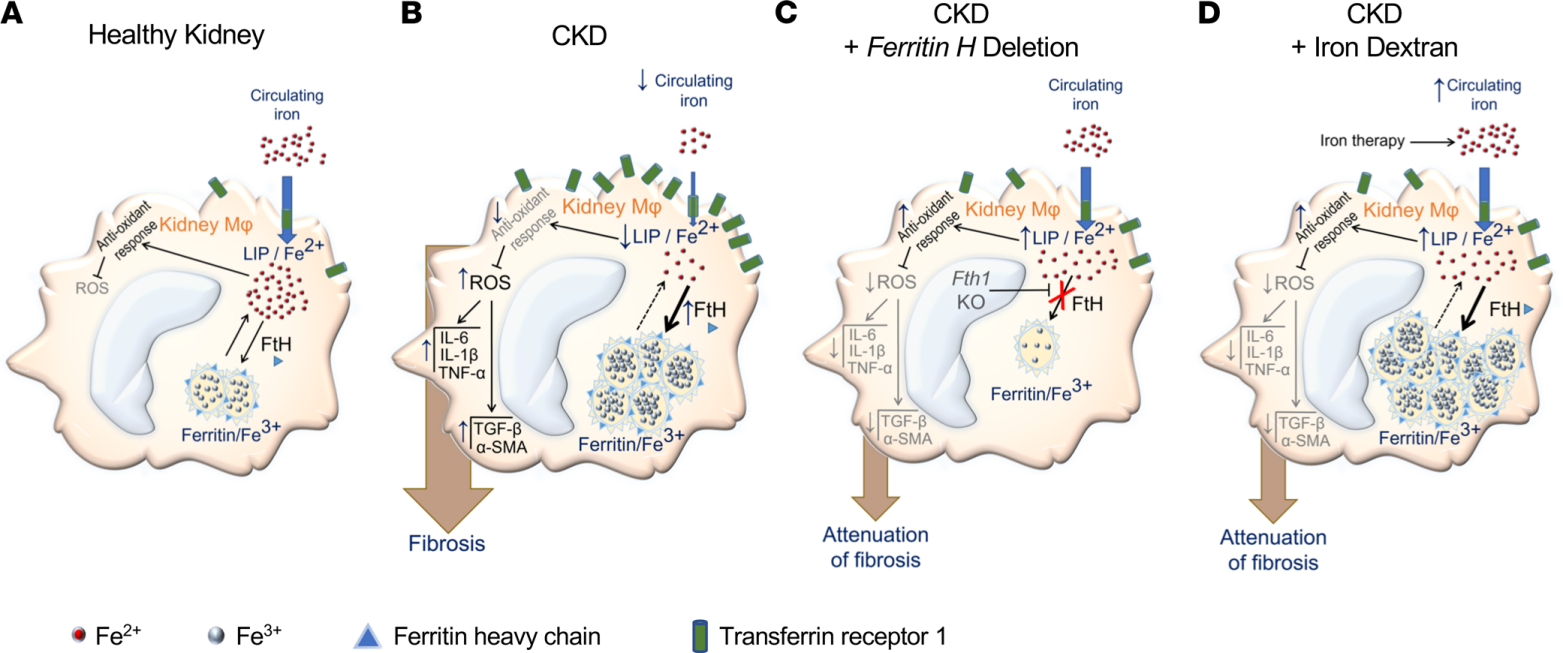
Proposed mechanism of the effects of kidney macrophage iron status in kidney fibrosis. (**A**) In healthy kidney macrophages, a balance between LIP (ferrous iron, Fe^2+^) and iron stores (ferritin-bound ferric iron, Fe^3+^) supports an adequate antioxidant response to oxidative stress and minimizes unwanted effects of ROS. (**B**) In kidney fibrosis, LIP of kidney macrophages, essential for the antioxidant response, is depleted. Consecutively, increased production of ROS, proinflammatory cytokines, and TGF-β mediates the profibrotic effects in kidney macrophages. (**C**) Blockade of FtH impairs conversion of Fe^2+^ to Fe^3+^ owing to the ferroxidase activity of FtH and replenishes LIP of kidney macrophages, which leads to reduced oxidative stress, reduced inflammation, and ultimately attenuation of fibrosis. (**D**) Similar to FtH blockade, increased iron supply via therapeutic iron administration replenishes LIP in kidney macrophages and reduces macrophage oxidative stress, inflammation, and production of TGF-β, which attenuates fibrosis.

In this study, we identified depletion of LIP in kidney macrophages as a driver of their pro-oxidative, proinflammatory, and profibrotic responses in CKD (Figure 9). Furthermore, we demonstrated that replenishment of intracellular iron levels in kidney macrophages by either therapeutic administration of iron dextran or myeloid lineage–specific deletion of *Fth1* attenuated kidney fibrosis. Thus, correction of intracellular iron deficiency of kidney macrophages appears as a promising therapeutic strategy to alleviate kidney fibrosis and slow CKD progression. We (8) and others (9, 10) have recently reported that selected iron preparations attenuate progression of experimental CKD. This was in some contrast with earlier studies that highlighted potential tubular toxicity of iron (24, 25). However, tubular cytotoxicity profile varies greatly depending on the type of iron formulation. While iron sucrose or iron gluconate has potent cytotoxicity, and could induce proximal tubular injury, iron dextran or iron oligosaccharide does not exhibit tubular nephrotoxicity (26). In the present study, weekly injections of iron dextran attenuated disease progression in 2 mouse models of kidney fibrosis. A similar improvement in kidney function was previously reported after a single injection of iron dextran in the 5/6-nephrectomy rat model of CKD (27).

Macrophages play an important role in kidney fibrosis (12) and, at the same time, are critical for the maintenance of systemic iron homeostasis (18), which is severely altered in CKD (6, 28). Our analysis demonstrated that in CKD, kidney macrophages develop intracellular iron deficiency, as indicated by the depletion of LIP and overexpression of TfR1. Hepcidin-induced systemic hypoferremia and elevation of myeloid FtH likely both contributed to LIP depletion in kidney macrophages in CKD. Importantly, LIP depletion was associated with induction of ROS and increased production of proinflammatory cytokines, TGF-β, and α-SMA by kidney macrophages, suggesting that macrophage iron deficiency is a major driver of proinflammatory and profibrotic behavior of these cells in CKD. Interestingly, kidney macrophages that express myofibroblast markers like α-SMA were recently shown to play an important signaling role in kidney fibrosis, while they may not be producing matrix (29).

Recent literature has challenged the outdated concept of cellular LIP being always directly associated with oxidative stress (30). CKD is a pro-oxidative condition and oxidative stress has been implicated in disease progression in patients with CKD (31, 32). While it is well known that iron excess can induce oxidative stress, iron deficiency leads to oxidative stress as well (33). The presence of anemia and iron deficiency increases the risk of oxidative stress in patients with CKD (34, 35). This phenomenon can be explained by the essential role of iron as a cofactor in many antioxidant enzymes (e.g., catalase). Our data suggest that the reduced LIP promotes oxidative stress in kidney macrophages by blunting their antioxidant response, either through impaired function of iron-containing antioxidant enzymes or due to limited transcriptional induction of antioxidant genes (35). In line with this, our kidney RNA sequencing demonstrated suppression of genes involved in antioxidant pathways in CKD mice. We and others have previously reported that ROS are the major mediators of macrophage inflammatory activation, which could explain the proinflammatory profile of kidney macrophages in CKD (36, 37). Indeed, treatment with the antioxidant vitamin E in our CKD model attenuated macrophage proinflammatory activation with beneficial effects on the kidney function.

To better understand the mechanistic role of depleted kidney macrophage LIP in kidney fibrosis, we used myeloid *Fth1* deletion to replenish macrophage LIP, without expanding ferritin-bound iron stores. The lack of the ferroxidase activity of FtH suppressed the accumulation of iron in ferritin, attenuating LIP depletion. This resulted in the attenuation of macrophage-derived proinflammatory and profibrotic responses and thus, an overall improvement of kidney function and fibrosis, which is further ameliorated by the combination with iron therapy. This was consistent with the study of Bolisetty et al., where myeloid *Fth1* deletion ameliorated acute kidney injury to CKD progression (38). Importantly, these observations suggest that the maintenance of physiological LIP levels in kidney macrophages, and not the stored (ferritin-bound) iron, is crucial to ensure a proper antioxidant response in the pro-oxidant CKD milieu, which is otherwise compromised when the regulatory LIP is depleted. Accordingly, myeloid FtH activity appears to be inappropriately elevated in the presence of reduced LIP, and deletion of *Fth1* in macrophages may have a therapeutic benefit in CKD.

The ability of iron to alter macrophage behavior in non-CKD settings is well established, though specific effects of iron on macrophage phenotype are largely determined by the microenvironment and the specific iron source (39). In hemolytic conditions, heme/iron-activated proinflammatory macrophages exert profibrotic and antihealing actions (36, 37, 40). In the tumor microenvironment, macrophages with protumor antiinflammatory properties are skewed by iron toward a proinflammatory phenotype with antitumoral activity (41–43). Iron accumulation in macrophages also plays a role in atherosclerosis, through excessive VEGF production and plaque instability (44, 45). In contrast, the present work shows that iron therapy alleviates rather than aggravates the proinflammatory and profibrotic skewing of kidney macrophages induced by CKD, in apparent contrast with previous observations of proinflammatory effects of iron in macrophages in other conditions. The observation that CKD is associated with intracellular iron deficiency in kidney macrophages reconciles these potentially novel findings with the previously described proinflammatory role of iron on macrophages in other pathologic conditions. In CKD, therapeutic iron dextran administration replenishes the depleted intracellular LIP, which in turn induces the antioxidant response, reduces ROS levels and inflammation, and ultimately attenuates kidney fibrosis. We also showed that iron exposure dampens the profibrotic response of macrophages to TGF-β stimulation, adding mechanistic insight into the antifibrotic action of iron therapy. Furthermore, our adoptive transfer of iron-loaded macrophages demonstrated the therapeutic potential of these cells for kidney fibrosis. This was in line with the recent report of Vaugier et al. showing that transfer of iron-loaded macrophages protected mice from ischemia/reperfusion injury (46).

Our RNA-sequencing data showed a significantly increased expression of numerous antioxidant genes in the kidney following iron therapy in CKD. For example, iron therapy upregulated *Gstm1*, a gene deficiency of which was recently linked to CKD progression in mouse models (47) and in large human cohorts (48, 49). Interestingly, while iron is traditionally regarded as a therapeutic agent with pro-oxidative potential (largely based on the assessment of oxidative status shortly after iron administration rather than long-term effects), several other studies in addition to ours documented improvement of oxidative stress following iron therapy in CKD. Indeed, Nuhu et al. showed that i.v. iron therapy with ferumoxytol improves systemic glutathione peroxidase activity in 5/6-nephrectomized rats (50). In the recent randomized IRON-CKD trial, systemic oxidative stress measured as thiobarbituric acid–reactive substances was reduced 1 month after injection of iron dextran or ferric derisomaltose in patients with predialysis CKD (51). These effects are likely associated with the ability of iron to boost the antioxidant response when it is suppressed or defective, such as in the setting of intracellular LIP deficiency.

Taken together, our data suggest that both systemic hypoferremia and ferritin induction in CKD contribute to the depletion of LIP in macrophages, resulting in their profibrotic activation. Interestingly, while iron therapy partially corrected macrophage LIP, it also resulted in a marked enlargement of the metabolically inactive ferritin-bound iron pool. This suggests that the pathologic activation of ferritin in CKD likely contributes to LIP reduction in favor of iron storage, which expands our understanding of the limited efficacy of systemic iron therapy in CKD, and calls for novel therapeutic strategies, possibly targeting the disrupted balance between labile and stored intracellular iron. It would also be of interest to assess the effect of other modalities of iron therapy, including oral iron agents commonly used in patients with CKD on kidney macrophage function and kidney fibrosis.

Our findings also unveil a new level of regulation of macrophage polarization status by iron, specifically by the LIP. In CKD, depletion of macrophage LIP triggers a defective antioxidant response and ROS accumulation, which likely contributes to cell switching toward a proinflammatory phenotype. Thus, our previous (36, 37) and current data support the concept that both extremes of intracellular iron balance, iron overload and deficiency (high and low LIP, respectively), induce macrophage inflammatory skewing through excessive ROS formation, in the first condition due to excess of catalytic iron, and in the second due to inadequate antioxidant response. Based on these findings, an evaluation of the effects that various iron preparations/regimens may have on kidney fibrosis, disease progression, and macrophage behavior in patients with CKD is warranted.

Several novel erythropoiesis-independent implications of iron therapy in CKD have emerged in recent years (6). Our study highlights a potentially novel “off-target” effect of parenteral iron therapy in CKD, which can be exploited as a therapeutic opportunity to slow disease progression. Specifically, our findings demonstrate that parenteral administration of iron dextran has disease-modifying properties and slows the progression of experimental CKD. This is mediated by repletion of macrophage intracellular LIP, which decreases oxidative stress and production of proinflammatory cytokines and TGF-β, thus alleviating kidney fibrosis.

## Methods

### Mice and CKD model.

Mice were housed and bred in the Weill Cornell Medicine animal facility. C57BL/6 mice were purchased from The Jackson Laboratory. Mice were fed 0.2% adenine diet (Envigo Teklad TD.140290) from 8 to 16 weeks of age to induce CKD as previously described (8). This model induces CKD and renal anemia that closely resemble changes in patients with CKD (52, 53). Wild-type mice were randomly assigned to the following groups: controls, CKD with no treatment, and CKD receiving i.p. iron dextran [a complex of ferric hydroxide, Fe(OH)_3_ and low molecular–weight (10,000) dextran, MilliporeSigma D8517] weekly, 0.5 g/kg/dose. Control mice received a diet identical to CKD mice but without adenine (Envigo Teklad TD.130898). Control and iron-untreated CKD mice received weekly i.p. injections of PBS. A separate group of mice received i.p. injections of α-tocopherol (vitamin E) 200 mg/kg every third day throughout the experimental period. At the end of the experimental period, mice were euthanized via pentobarbital injection.

*Fth1*^fl/fl^ mice (The Jackson Laboratory stock 018063) were bred with LysM-Cre mice (The Jackson Laboratory stock 004781) to generate LysM-Cre^+^ *Fth1*^fl/fl^ (*Fth1*^LysM+/–^) and LysM-Cre^–^ *Fth1*^fl/fl^ (*Fth1*^LysM–/–^) littermates, provided to us by Suzanne Cloonan and Bill Zhang (Weill Cornell Medicine, New York, New York, USA). Ablation of Fth1 in myeloid cells was confirmed by Western blot of BMDMs obtained from *Fth1*^LysM+/–^ mice. CKD was induced in *Fth1*^LysM+/–^ and *Fth1*^LysM–/–^ mice with adenine similar to wild-type mice. A subgroup of *Fth1*^LysM+/–^ mice received weekly i.p. injections of iron dextran, 50 mg/kg/dose. This iron dextran dose was lower than the dose used in experiments employing only wild-type mice. The dose of iron dextran used in iron therapy experiments utilizing only wild-type mice was lethal in *Fth1*^LysM+/–^ mice.

Mice expressing CD45.1 allele were purchased from The Jackson Laboratory (stock 002014) and used for macrophage adoptive transfer experiments. Wild-type C57BL/6 strains expressed the CD45.2 allele.

### Surgical protocol.

UUO was performed as previously described (17). In brief, mice were anesthetized by an i.p. injection of ketamine/xylazine solution (100:10 mg/kg). Mice then were subjected to a dorsal lumbar incision; the left ureter was isolated and ligated 3–5 mm below its origin. Sham-operated mice had their ureters exposed but not ligated. The flank incision was closed with 4-0 silk sutures. In 7 days, mice were re-anesthetized for kidney harvesting and euthanasia.

### Biochemistry and blood counts.

Blood was collected from the inferior vena cava at euthanasia. BUN was measured on the Beckman Coulter AU 680 analyzer. Blood counts were performed on the IDEXX Procyte DX analyzer. Both procedures were performed with the assistance of the Weill Cornell Medicine Laboratory of Comparative Pathology. Serum iron levels were measured using SFBC kits (Biolabo) following manufacturer’s instructions. We used ELISA to measure serum cystatin C (R&D Systems, Bio-Techne), serum soluble TfR (MyBioSource), and urine albumin (Abcam) following manufacturers’ instructions.

### Histology and immunohistochemistry.

Mouse kidney and spleen samples were fixed in 4% paraformaldehyde (Santa Cruz Biotechnology) overnight. After washing in fresh PBS, fixed tissues were dehydrated, cleared, and embedded in paraffin. Paraffin blocks were cut into 5 μm thick sections, which were placed on positively charged slides, deparaffinized, and stained with Masson’s trichrome to assess collagen deposition in kidney fibrosis and Perls Prussian blue to visualize ferric iron. Kidney fibrosis was quantified as percentage of midsagittal kidney sections affected by interstitial fibrosis. Immunofluorescence staining was performed on frozen kidney sections using BM8 anti-F4/80 (eBioscience, Thermo Fisher Scientific, 14-4801-82) and anti-CD45.1 (Abcam ab25348) antibody. Immunohistochemistry staining using the same anti-F4/80 antibodies of 4% paraformaldehyde-fixed, paraffin-embedded kidney samples was performed using standard protocols as previously described (54). Briefly, sections were subjected to steam boiling (catalog IW-1102; IHC World) for 30 minutes in antigen retrieval buffer (pH 6.0). Slides were incubated with blocking buffer, followed by Avidin D and Biotin blocking reagent (Vector Laboratories). Kidney sections with anti-F4/80 antibodies were incubated overnight at 4°C. Sections were developed with Vectastain ABC kit (Vector Laboratories PK4001) followed by hematoxylin staining (Vector Laboratories H3401).

### Transmission electron microscopy.

Kidney tissues were fixed with 2.5% glutaraldehyde (MilliporeSigma), 4% paraformaldehyde (Santa Cruz Biotechnology Inc.), and 0.02% picric acid in 0.1 M sodium cacodylate buffer (MilliporeSigma) at pH 7.2 for overnight at 4°C. Tissues were washed twice with 0.1 M sodium cacodylate buffer. Following a secondary fixation in 1% osmium tetroxide and 1.5% potassium ferrocyanide (MilliporeSigma), the samples were dehydrated through a series of graded ethanol (Thermo Fisher Scientific) and embedded in an EPON analog resin (MilliporeSigma). Ultrathin sections were cut using Accu-Edge high-profile blades (Sakura, 4685) on a Microm HM 355S (Thermo Fisher Scientific). Sections were placed on the copper grids, contrasted with lead citrate, and captured on the electron microscope (JEM 1400, JEOL). Images were recorded with the associated digital camera (Veleta 2K, Olympus-SIS).

### Western blot.

Kidney tissues and BMDMs were harvested in tissue extraction (Thermo Fisher Scientific, 78510) and mammalian protein extraction (Thermo Fisher Scientific, 78501) buffers, respectively, containing protease inhibitor cocktail (Thermo Fisher Scientific, 78441). Kidney mononuclear cells were harvested in RIPA buffer (Thermo Fisher Scientific 89901). Protein concentrations were determined using the BCA assay (Thermo Fisher Scientific 78441). Protein extracts (both cellular and tissue homogenates) were subjected to SDS-PAGE separation on a 10% Criterion TGX Precast Gels gel electrophoretic system (Bio-Rad). Proteins were electroblotted onto PVDF membranes (Bio-Rad). After transfer, nonspecific binding was prevented using 5% skim milk (Santa Cruz Biotechnology) or 5% BSA in 0.1% TBS-Tween (Bio-Rad). The blots were incubated with anti-fibronectin (Abcam ab2413), anti-ferritin heavy chain 1 (Cell Signaling Technology 3998 and Abcam Ab183781), anti–α–SMA (Abcam ab5694), anti–TGF-β (Cell Signaling Technology 3711), anti-F4/80 (sc-377009), anti-iNOS (Abcam ab3523), anti-GAPDH (Cell Signaling Technology 2118), and anti–β-actin (MilliporeSigma A2228) overnight at 4°C. The blots were then washed and incubated for 1 hour, at room temperature, with individual secondary antibodies (anti-rabbit IgG HRP-linked antibody, Cell Signaling Technology 7074S, and anti-mouse IgG HRP-linked antibody, Cell Signaling Technology 7076S) accordingly. Bands were detected using an enhanced chemiluminescence detection system (Bio-Rad). The detected bands were quantified by densitometry through ImageJ 1.41 software (NIH).

### RNA isolation and RNA sequencing.

Total RNA was extracted from whole kidney tissues using RNeasy Micro Kit (catalog number 74004; QIAGEN) according to the manufacturer’s instructions. Following RNA isolation, total RNA integrity was checked using a 2100 Bioanalyzer (Agilent Technologies). RNA concentration was measured using the NanoDrop system (Thermo Fisher Scientific). Preparation of RNA sample library and RNA sequencing were performed by the Genomics Resources Core Facility at Weill Cornell Medicine. mRNA was prepared using TruSeq Stranded mRNA Sample Library Preparation kit (Illumina). The normalized cDNA libraries were pooled and sequenced on an Illumina HiSeq4000 sequencer with single-end 50 cycles and analyzed using STAR (V2.5.2) and DESeq2 package. Normalized read counts were tested with DESeq for differential gene expression against control samples using an adjusted *P* value cutoff of 0.5. RNA-sequencing data were deposited into the NCBI’s Gene Expression Omnibus database (accession number GSE221598).

### Cell culture and adoptive transfer of macrophages.

Bone marrow cells from C57BL/6 wild-type, *Fth1*^LysM+/–^, and CD45.1^+^ mutant mice were isolated from femurs and differentiated into macrophages after culturing at 10^6^ cells/mL for 7 days in DMEM (MilliporeSigma) supplemented with 10 mM glutamine, 25 mM HEPES, 100 U/mL penicillin, and 100 μg/mL streptomycin (all from Gibco, Thermo Fisher Scientific), plus 10% fetal bovine serum (Thermo Fisher Scientific) and monocyte colony-stimulating factor (10 ng/mL; Stemcell Technologies). After differentiation, cells were treated with TGF-β1 (5 ng/mL; R&D Systems, Bio-Techne) with and without ferric ammonium citrate (25 μM, MilliporeSigma) for 20 hours. Cells were then processed for protein extraction and Western blot.

In separate experiments, BMDMs obtained from CD45.1^+^ mice (with and without iron loading) were administered i.v. by retro-orbital injection (5 × 10^6^ cells per mouse) to the wild-type (CD45.2^+^) mice 1 hour after UUO.

### Flow cytometry.

Kidney macrophages were analyzed for levels of cytokines, ROS, and LIP by flow cytometry. Kidneys were digested with a buffer containing collagenase and mechanically disrupted. The cell suspension was forced through a 100 μm cell strainer (Falcon, Corning) and depleted of red blood cells (RBC) with RBC lysis solution (Thermo Fisher Scientific) when needed. Afterward, the cell suspension was centrifuged at 1,700 rpm 5 minutes at 4°C and stained for flow cytometry. Cells were incubated with Fc-γ receptor blocking solution (CD16/32) (Invitrogen, Thermo Fisher Scientific, MFCR004) and stained with antibodies against CD45, Ly6G, CD11b, CD11c, MHCII, CD64, and CD24 and live/dead stain 7AAD (BioLegend). Kidney macrophages were identified among CD45^+^Ly6G^–^ cells as CD11b^+^CD64^+^CD24^–^. For surface staining, live cells were stained with antibodies against CD71 (TfR1) and CD206 for 30 minutes at 4°C. For intracellular staining, cells previously stained for macrophage-specific surface markers, as described above, were fixed and permeabilized in perm/wash solution (eBioscience, Thermo Fisher Scientific) according to manufacturer’s instruction and then stained in Permeabilization Buffer (eBioscience, Thermo Fisher Scientific). Intracellular staining was performed using antibodies against IL-6, TNF-α, IL-1β, iNOS, and TGF-β. Cells unstained and/or stained with antibody isotypes were used as controls. For LIP and ROS staining, cells previously stained for macrophage-specific surface markers, as described above, were freshly stained with FerroOrange (MilliporeSigma) and CellROX (Thermo Fisher Scientific), respectively, for 30 minutes at room temperature. Data were acquired by FACSymphony flow cytometer (BD Biosciences), and analysis was performed using FlowJo software (BD Biosciences). The levels of cytokines, CD206, iNOS, LIP, and CD71 ROS are shown in MFI as a fold change to the control condition.

Antibodies and fluorochromes used for the analysis of kidney macrophages are shown in Supplemental Table 2.

### Statistics.

Data were reported as mean ± SEM except where noted. One-way ANOVA was used for the analysis of differences among more than 2 groups with Tukey’s post hoc test. The differences between 2 groups were assessed with 2-tailed *t* test unless specified. *P* values less than 0.05 were considered statistically significant. GraphPad Prism was used for statistical analyses.

### Study approval.

All animal studies were carried out following the NIH *Guide for the Care and Use of Laboratory Animals* (National Academies Press, 2011) and were approved by the Institutional Animal Care and Use Committee of Weill Cornell Medicine.

## Author contributions

OA, FV, DB, and MEC conceived and designed the studies; EP, DB, SZV, AA, RU, CC, CGC, and SJ conducted the experiments; EP, CC, OA, FV, and DB analyzed the data; EP, OA, and FV drafted the manuscript; DB, OA, FV, and MEC edited and revised the manuscript; and all authors approved the final manuscript.

## Supplementary Material

Supplemental data

## Version 1 11/17/2022

In-Press Preview

## Version 2 01/10/2023

Electronic publication
