# Supplementary material for: Iron therapy mitigates chronic kidney disease progression by regulating intracellular iron status of kidney macrophages
Source: JCI Insight. 2023 Jan 10;8(1):e159235. doi: 10.1172/jci.insight.159235 (PMC9870080; doi:10.1172/jci.insight.159235)
Supplement: Supplemental data [file jciinsight-8-159235-s264.pdf]

Iron therapy mitigates chronic kidney disease progression by regulating intracellular iron status of kidney macrophages

Edwin Patino, Divya Bhatia, Steven Z. Vance, Ada Antypiuk, Rie Uni, Chantalle Campbell, Carlo Castillo, Shahd Jaouni, Francesca Vinchi, Mary E. Choi, Oleh Akchurin

SUPPLEMENTAL MATERIAL

Supplemental Table 1. Hematologic parameters in CKD mice in the presence and absence of iron therapy and in control mice

	CTR (n=9)	AD (n=9)	AD Fe (n=9)
Hematocrit (%)	49.5 ± 2.9^{ab}	33.2 ± 1.6^{ac}	38.9 ± 2.3^{bc}
Red blood cell count (M/uL)	10.13 ± 0.53^{ab}	8.6 ± 0.7^{a}	8.8 ± 1.8^{b}
MCV (fL)	50.1 ± 1.4^{ab}	39.7 ± 0.9^{ac}	48.1 ± 1.0^{bc}
MCH (pg)	14.7 ± 0.4^{a}	11.6 ± 0.3^{ac}	14.8 ± 0.2^{c}
MCHC (g/dL)	29.3 ± 0.6^{b}	29.3 ± 0.4^{c}	30.8 ± 0.5^{bc}
RDW-CV (%)	24.8 ± 1.2^{ab}	27.7 ± 1.0^{ac}	23.4 ± 0.9^{bc}
Reticulocyte count (K/uL)	439.3 ± 80.5^{a}	673.8 ± 122.7^{ac}	440.4 ± 140.8^{c}
Platelets (K/uL)	691.3 ± 249.1^{ab}	1688 ± 228.3^{ac}	1277 ± 258.7^{bc}
WBC (K/uL)	4.6 ± 3.7	2.3 ± 0.7	2.9 ± 1.7
Neutrophil (%)	17.4 ± 6.9^{ab}	40.1 ± 11.9^{a}	44.7 ± 9.6^{b}
Lymphocyte (%)	77.5 ± 8.1^{ab}	50.3 ± 12.8^{a}	45.5 ± 9.1^{b}
Neutrophil / lymphocyte ratio	0.2 ± 0.1^{ab}	0.9 ± 0.6^{a}	1.1 ± 0.4^{b}
Monocyte (%)	2.1 ± 2.0^{ab}	6.5 ± 2.4^{a}	6.9 ± 1.8^{b}

Differences between the groups were analyzed using ANOVA with the Tukey post hoc test.

[a] $p<0.05$ between CTR and AD

[b] $p<0.05$ between CTR and AD Fe

[c] $p<0.05$ between AD and AD Fe

Supplemental Table 2. Antibodies and fluorochromes used for the flow cytometry analysis of kidney macrophages.

Color	Antibody	Clone	Brand
PerCP	**7AAD**	#420404	Biolegend
Pacific Blue	**CD45**	104	Biolegend
BUV395	**Ly6G**	1A8	BD Horizon
APC-Cy7	**CD11b**	M1-70	Biolegend
APC	**CD11c**	HL3	BD Horizon
PE-Cy7	**MHCII**	M5/114.15.2	Biolegend
BV711	**CD64**	X54-5/7.1	Biolegend
BV605	**CD24**	M1/69	Biolegend
FITC	**CD206**	C068C2	Biolegend
PerCp-eFluor710	**CD71**	R17217	eBioscience
AF700	**TNFa**	MP6-XT22	Biolegend
PerP-eFluor710	**IL6**	MP5-20F3	Invitrogen
PE	**IL-1b**	NJTEN3	Invitrogen
FITC	**iNOS**	#610330	BD Horizon
FITC	**TGF-b**	TW7-16B4	Biolegend
AF700	**α-SMA**	1A4	R&D Systems
FITC	**ROS**	CellRox	ThermoFisher
PE	**LIP**	FerroOrange	Sigma-Aldrich
FITC/PE	**BODIPY™**	581/591 C11	Invitrogen

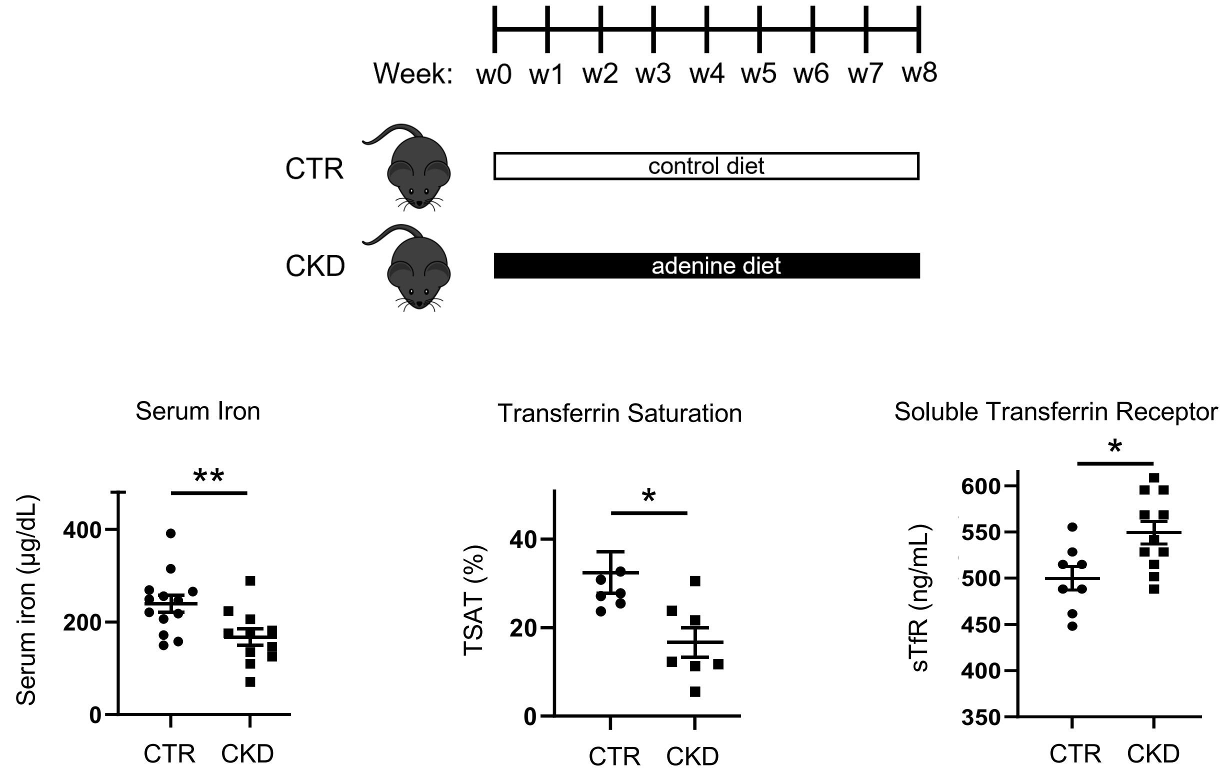

Supplemental Figure 1. Parameters of systemic iron status in mice with chronic kidney disease and in control mice. Blood was collected at euthanasia after 8 weeks of adenine or control diet. Serum iron, transferrin saturation (TSAT) and soluble transferrin receptor (sTfR) in two groups of mice (n=6-13 per group). Error bars represent SEM. Data were analyzed using *t* test; **P*<0.05, ***P*<0.01.

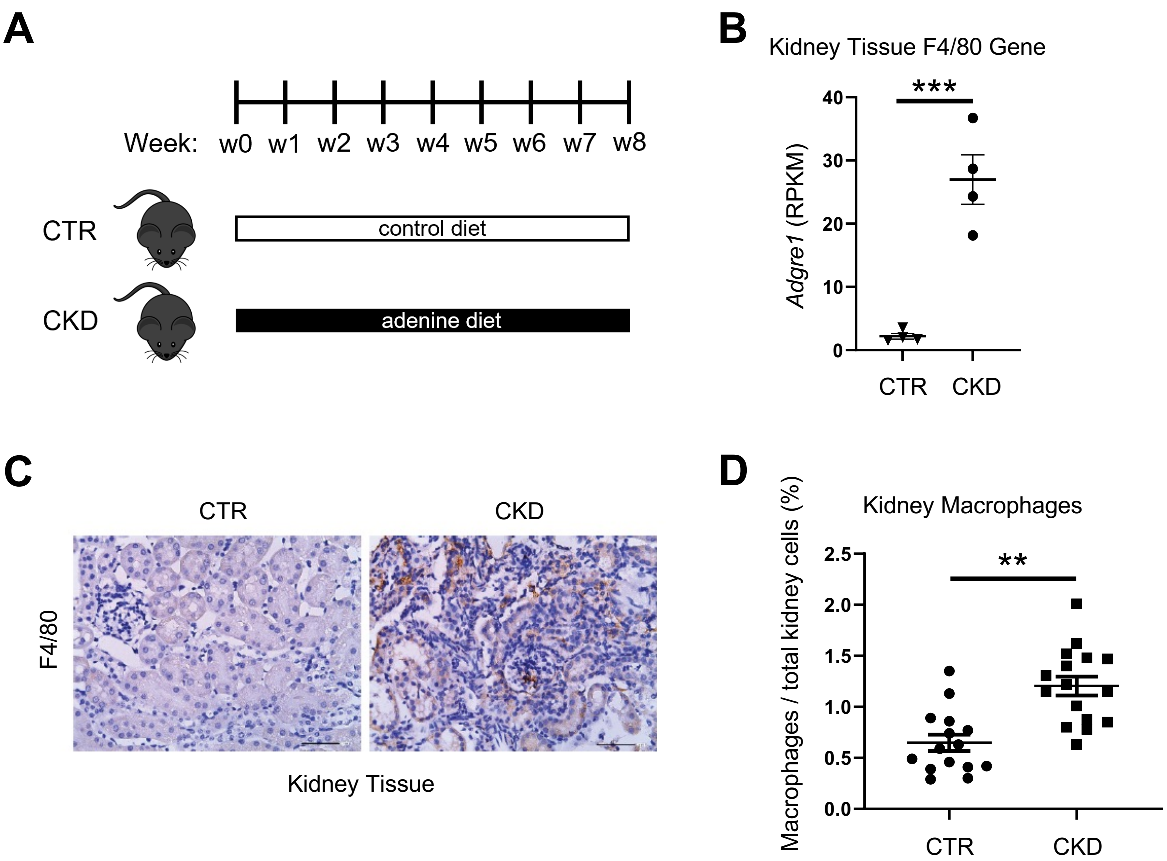

Supplemental Figure 2. Kidney macrophages in healthy control mice and in mice with chronic kidney disease (**A**) Schematic diagram of chronic kidney disease (CKD) induction by a 0.2% adenine diet for 8 weeks. (**B**) Macrophage marker F4/80 gene expression in whole kidney tissue (n=4 per group) and (**C**) F4/80 immunostaining in control (CTR) and CKD kidneys (scale bars, 50 μm). (**D**) Percent of macrophages by total kidney cell counts was assessed by flow cytometry of kidney lysates; n=15-16 per group. Error bars represent SEM. Data were analyzed using *t* test; *P<0.05, **P<0.01.

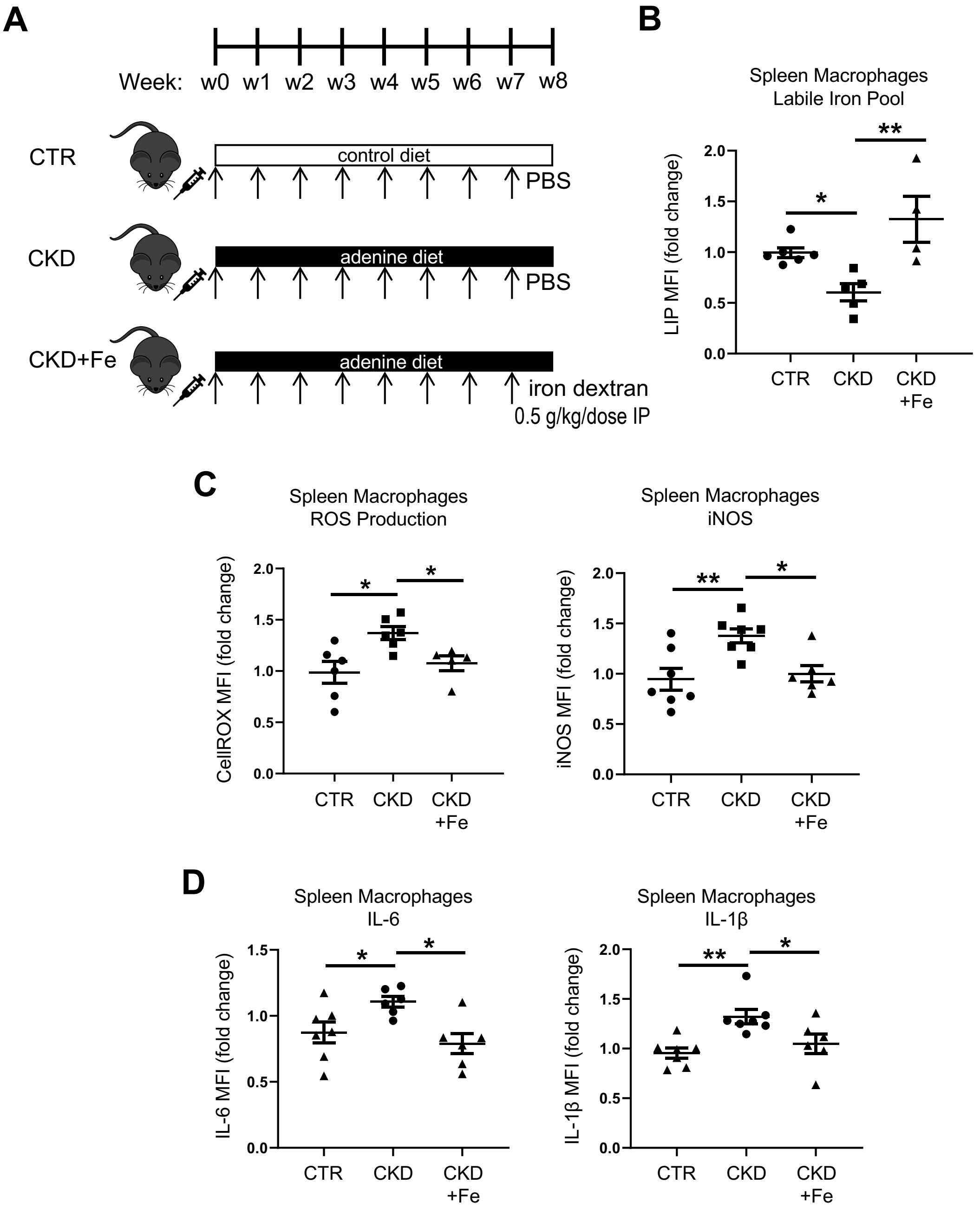

Supplemental Figure 3. Chronic kidney disease-induced polarization of spleen macrophages and effect of iron therapy. (**A**) Schematic diagram of CKD induction and iron therapy. Iron dextran was administered intraperitoneally once a week, 0.5 g/kg (CKD+Fe group) throughout experimental period. (**B**) Spleen macrophage labile iron pool (LIP) in CTR, CKD, and CKD+Fe groups. (**C**) Reactive oxygen species (ROS) production and iNOS expression in kidney macrophages in three groups of mice. (**D**) Production of pro-inflammatory cytokines IL-6 and IL-1β in kidney macrophages; CTR, CKD, and CKD+Fe groups. Error bars represent SEM, n=4-7 per group. Data were analyzed using ANOVA. *P<0.05; **P<0.01; ***P<0.001.

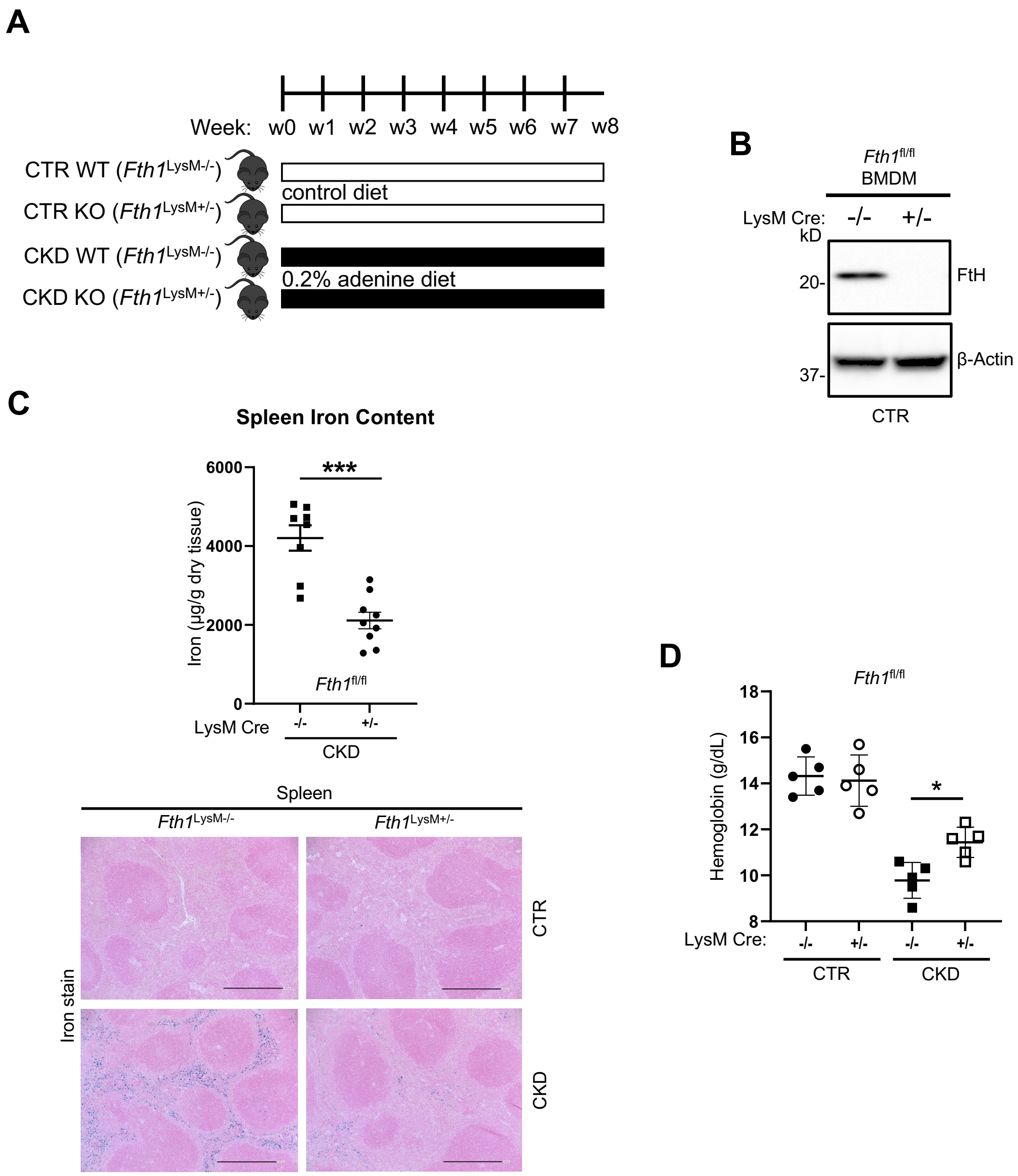

Supplemental Figure 4. Systemic hematologic effects of myeloid ferritin heavy chain deletion in mice with chronic kidney disease. (**A**) Chronic kidney disease (CKD) was induced by a 0.2% adenine which mice received for 8 weeks. Control (CTR) mice received non-adenine containing but otherwise identical diet. (**B**) Knockout (KO) of *Fth1* was confirmed by FtH protein immunoblotting of bone marrow derived macrophages (BMDM) obtained from *Fth1*$^{LysM-/-}$ (wild type, WT) and *Fth1*$^{LysM+/-}$ (KO) mice using EPR18878 (ab183781 antibody). (**C**) Assessment of spleen iron by dry tissue iron content measurement (n=8-9 per group) and histology (Perls stain; scale bars, 500 µm). (**D**) Myeloid-specific (LysM-Cre) *Fth1* deletion improved hemoglobin in CKD mice (n=5 per group). Blood was collected at euthanasia after 8 weeks of control or adenine diet. Data were analyzed using ANOVA (B) and *t* test (C). *P<0.05; ***P<0.001.

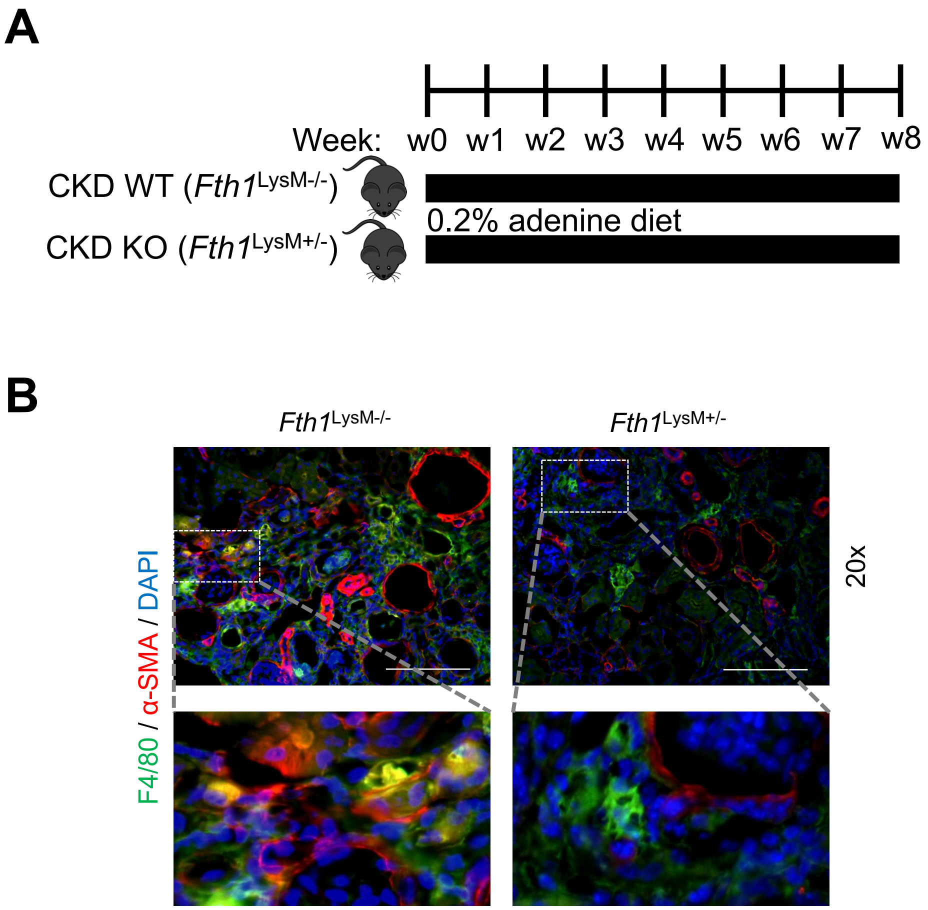

Supplemental Figure 5. Myeloid-specific deletion of FtH reduces kidney macrophage to myofibroblast transition in mice with chronic kidney disease. (**A**) Schematic diagram depicting the timeline of chronic kidney disease (CKD) induction in mice with myeloid-specific (LysM-Cre) deletion of ferritin heavy chain gene (*Fth1*) and wild type mice. (**B**) Immunostaining indicates reduced co-expression of macrophage marker F4/80 and myofibroblast marker α-smooth muscle actin (α-SMA) in kidney tissues of *Fth1*$^{LysM+/-}$ mice with CKD compared to *Fth1*$^{LysM-/-}$ mice with CKD. Scale bars, 200 μm.

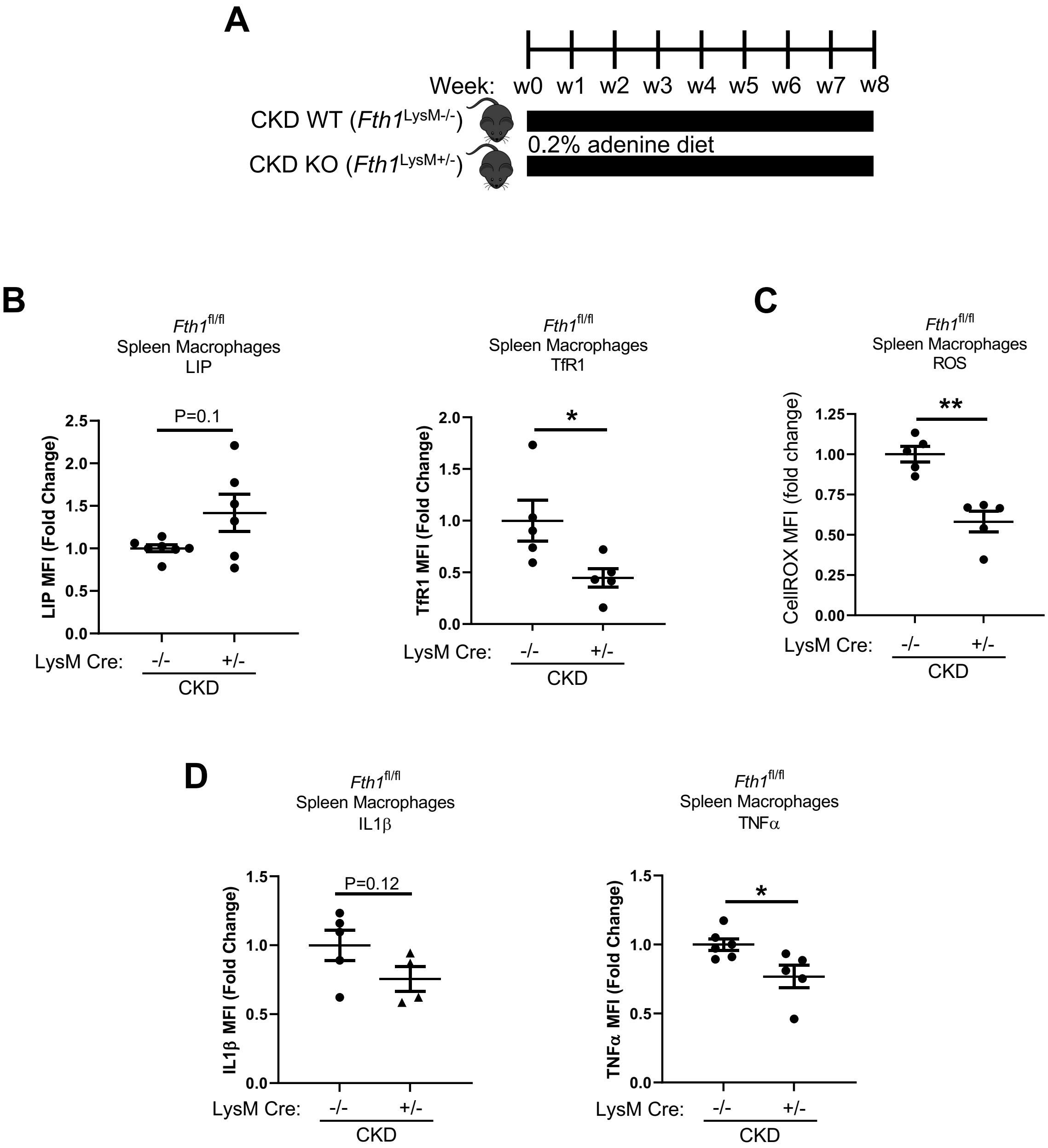

Supplemental Figure 6. Effects of myeloid ferritin heavy chain deletion on spleen macrophages in mice with chronic kidney disease. (**A**) Schematic diagram depicting induction of chronic kidney disease (CKD) in wild type mice and in mice harboring myeloid-specific (LysM-Cre) deletion of ferritin heavy chain gene (*Fth1*). (**B**) Markers of intracellular iron status of spleen macrophages, labile iron pool (LIP) and transferrin receptor 1 (TfR1) in two groups of mice. (**C**) Assessment of spleen macrophage oxidative stress in CKD *Fth1*$^{LysM-/-}$ and *Fth1*$^{LysM+/-}$ CKD mice. (**D**) Effect of *Fth1* deletion on pro-inflammatory cytokines IL-1β and TNF-α in spleen macrophages in CKD. Error bars represent SEM. Data were analyzed by *t* test, n=4-6 per group; **P*<0.05.

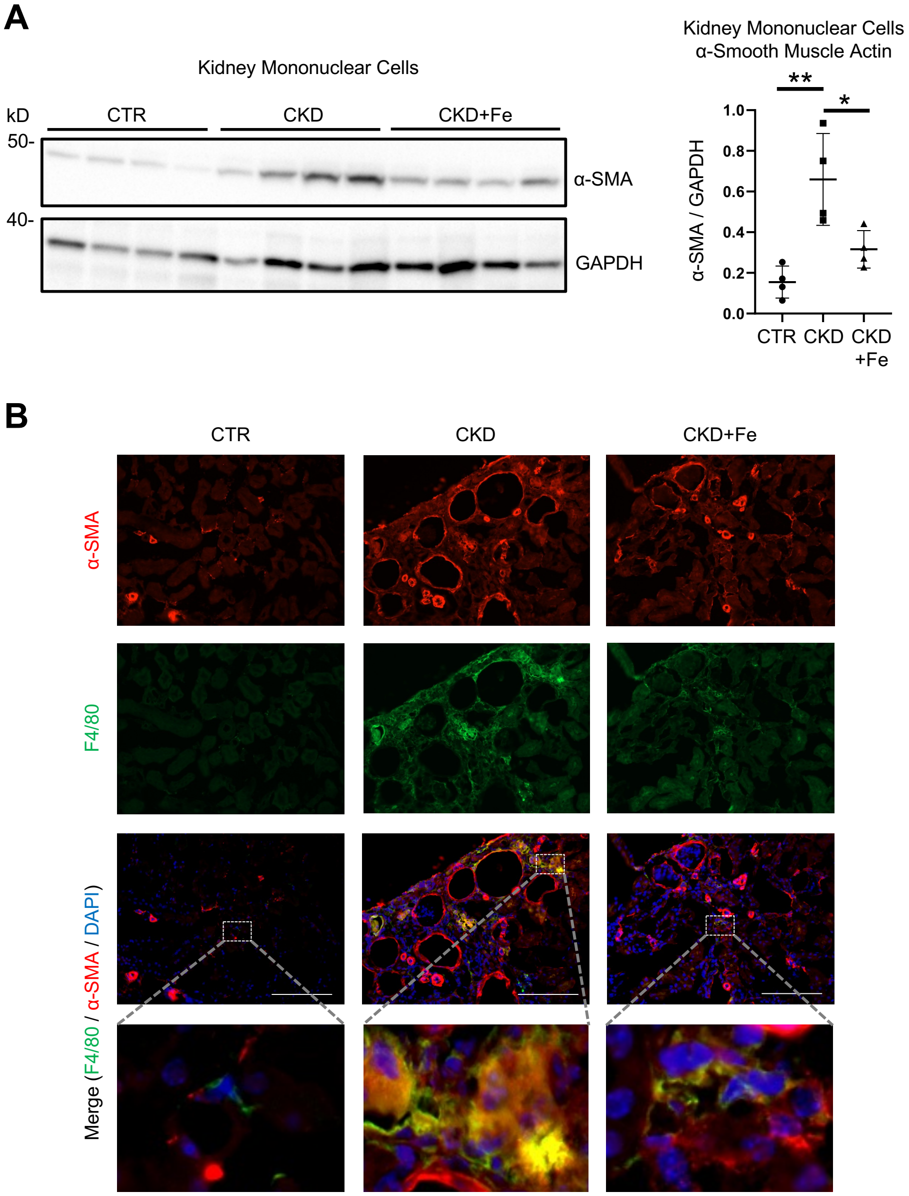

Supplemental Figure 7. Effect of iron dextran therapy on macrophage to myofibroblast transition in the kidneys of mice with chronic kidney disease. Chronic kidney disease **(**CKD) was induced by 0.2% adenine diet. Iron dextran was administered intraperitoneally once a week, 0.5 g/kg (CKD+Fe group). (**A**) Expression of α-smooth muscle actin in kidney mononuclear cells isolated from kidney single cell suspensions by Ficoll-Hypaque density gradient centrifugation (same membrane as used for Figure 1); n=4 per group. (**B**) Immunostaining for macrophage marker F4/80 and myofibroblast marker α-smooth muscle actin (α-SMA) demonstrates reduced colocalization between those markers in kidney tissues of CKD+Fe mice compared to the untreated CKD group. Scale bars, 200 μm (20x magnification). Error bars indicate SD. Densitometry data were analyzed by ANOVA; **P*<0.05; ***P*<0.01.

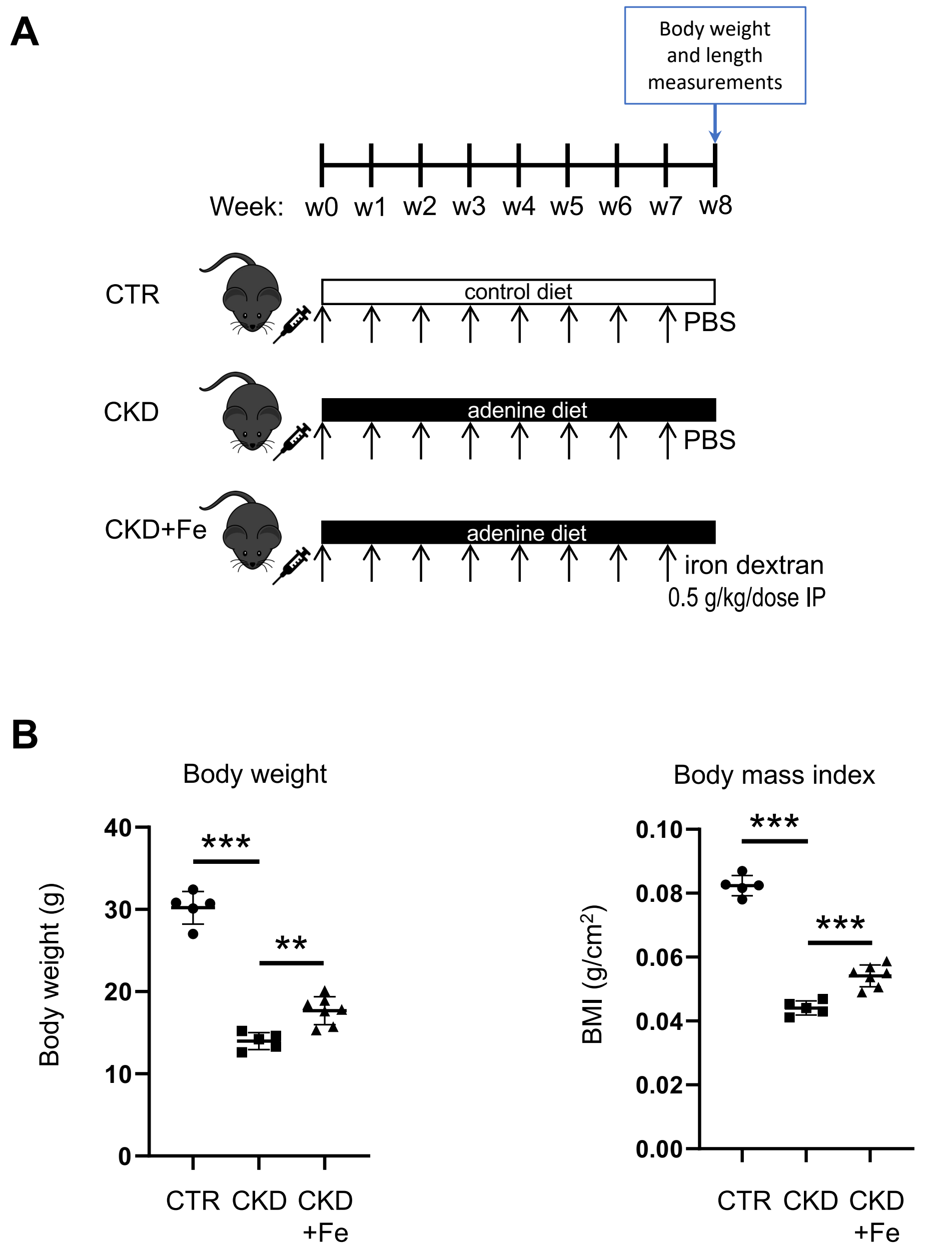

Supplemental Figure 8. Body measurements at the end of experimental period in control mice and in chronic kidney disease mice in the presence and absence of iron therapy. (**A**) Schematic diagram of chronic kidney disease (CKD) induction and iron therapy. Iron dextran was administered intraperitoneally once a week, 0.5 g/kg (CKD+Fe group) throughout experimental period. (**B**) Body weight and body mass index (BMI) in control (CTR), CKD, and CKD+Fe groups at euthanasia, n=5-7 per group. Data were analyzed by ANOVA. Error bars represent SD; ***P*<0.01; ****P*<0.001.

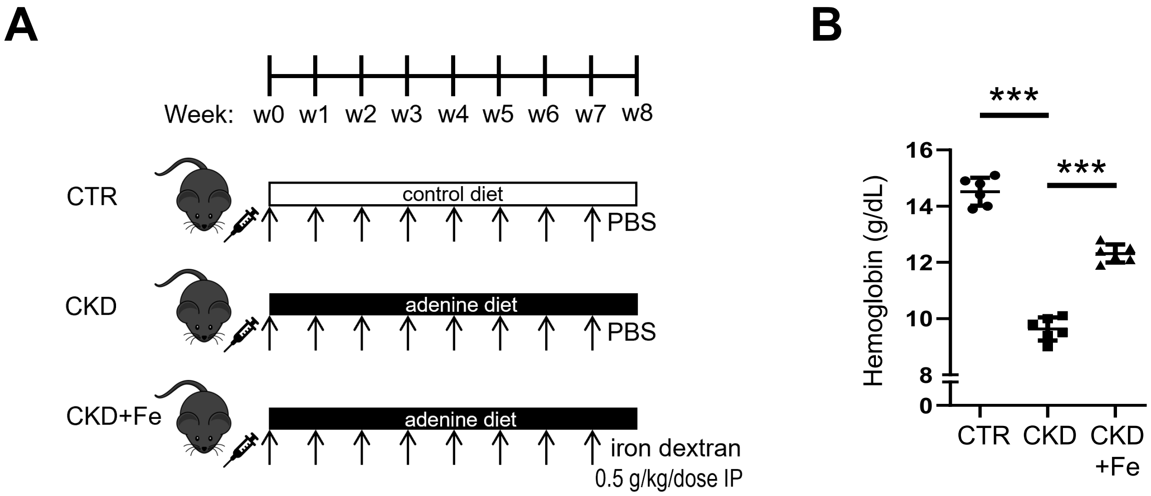

Supplemental Figure 9. Iron dextran therapy improves anemia in mice with adenine-induced CKD. (**A**) Schematic diagram of CKD induction and iron therapy. Iron dextran was administered intraperitoneally once a week, 0.5 g/kg (CKD+Fe group). Blood for hemoglobin measurements was collected at euthanasia (**B**) Mice with CKD developed anemia after 8 weeks of adenine diet. Iron therapy improved anemia in CKD mice. Data were analyzed by ANOVA, n=6 per group. Error bars represent SD; ****P*<0.001.

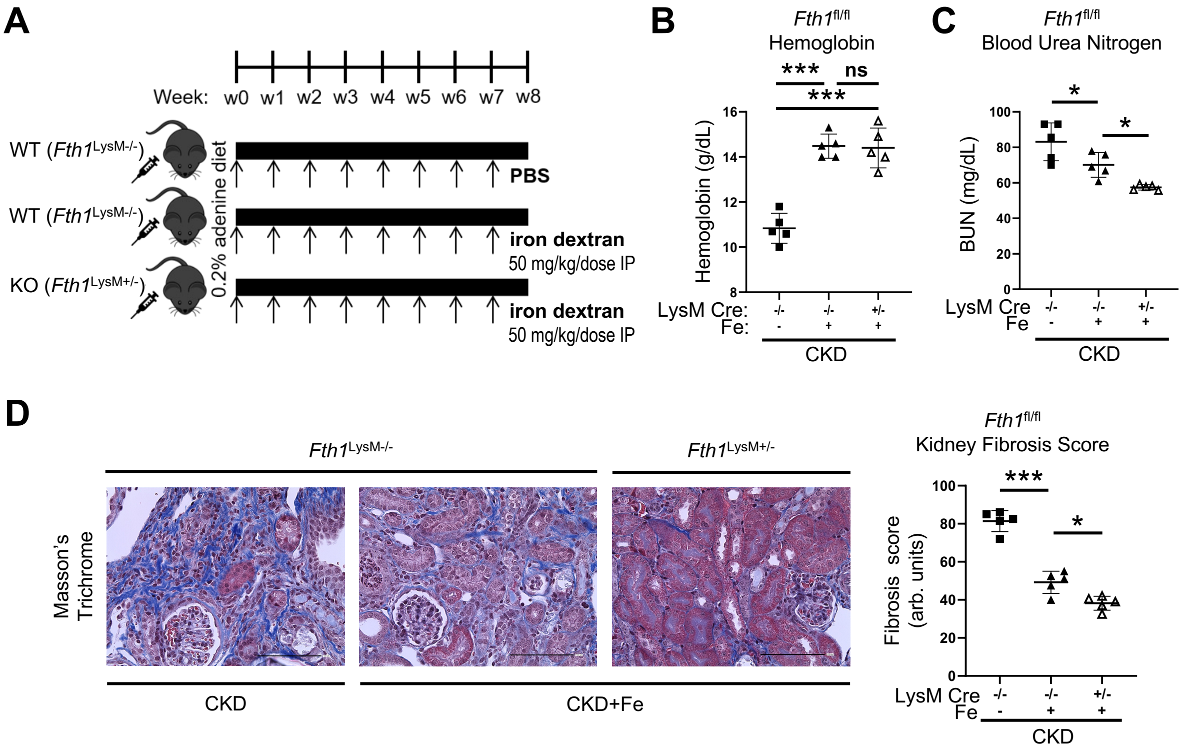

Supplemental Figure 10. Iron administration and *Fth1* deletion have additive protective effect against kidney fibrosis in mice with CKD. (**A**) Schematic diagram of the experimental groups: (1) wild type CKD mice, no iron therapy, (2) wild type CKD mice + iron therapy, (3) Myeloid-specific (LysM-cre) *Fth1* knockout CKD mice + iron therapy. Iron was administered via intraperitoneal injections of iron dextran, 50 mg/kg/dose. (**B**) Effect of iron administration on hemoglobin was similar in *Fth1*$^{LysM+/-}$ and *Fth1*$^{LysM-/-}$ CKD mice. (**C**) Blood urea nitrogen (BUN) in three groups of mice at the end of experimental period. (**D**) Assessment of kidney fibrosis in three groups of mice by Masson's trichrome staining, representative images and quantification. Scale bars, 100 μm. Error bars represent SD; n=5 per group. Data were analyzed using ANOVA. *$p<0.05$; ***$p<0.001$.

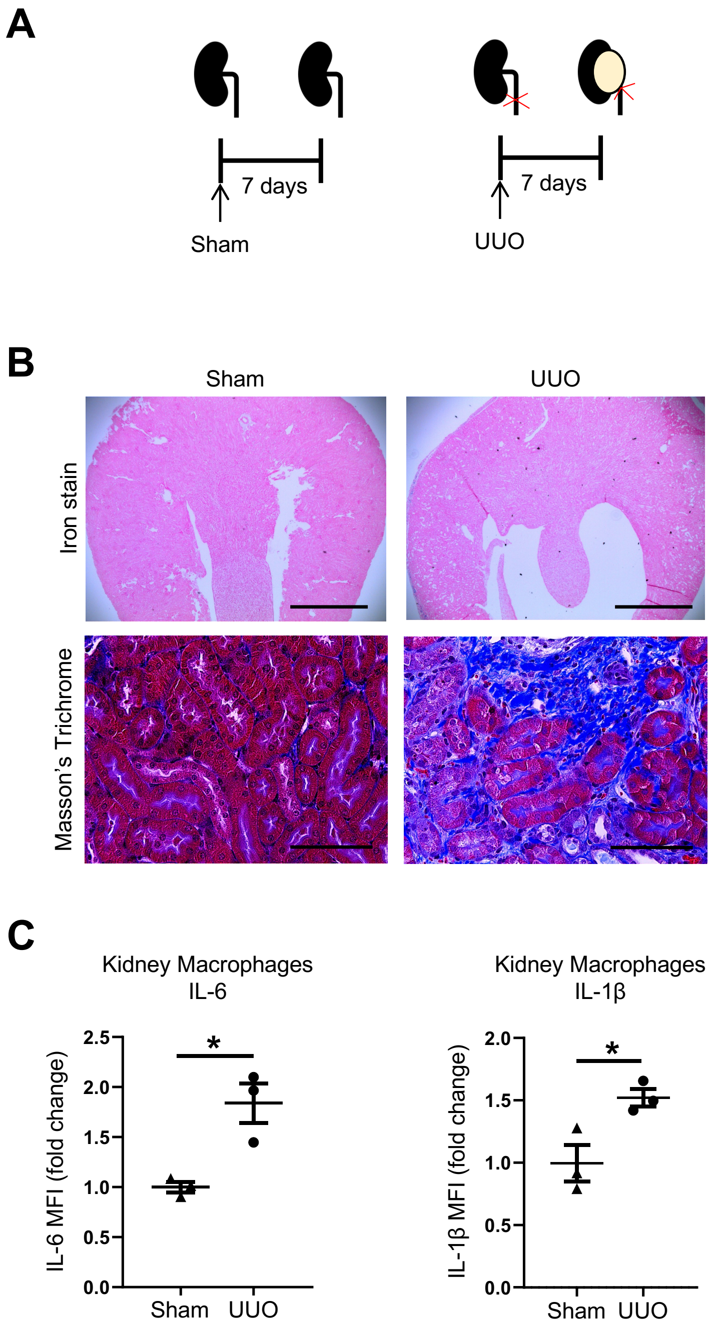

Supplemental Figure 11. Kidney macrophages are pro-inflammatory in the unilateral ureteral obstruction model of kidney fibrosis. (**A**) Experimental scheme of the unilateral ureteral obstruction (UUO) experiments. Kidneys were harvested 7 days after UUO or Sham surgery. (**B**) Perls' Prussian blue staining (upper panels; scale bars, 1.0 mm). Trichrome staining (lower panels; scale bars, 100 μm) demonstrating severe tubulointerstitial fibrosis in obstructed kidneys compared to Sham control (**C**) IL-6 and IL-1β production is induced in UUO kidney macrophages compared to Sham. Error bars represent SEM, n=3 per group. Data were analyzed using *t* test; **P*<0.05.

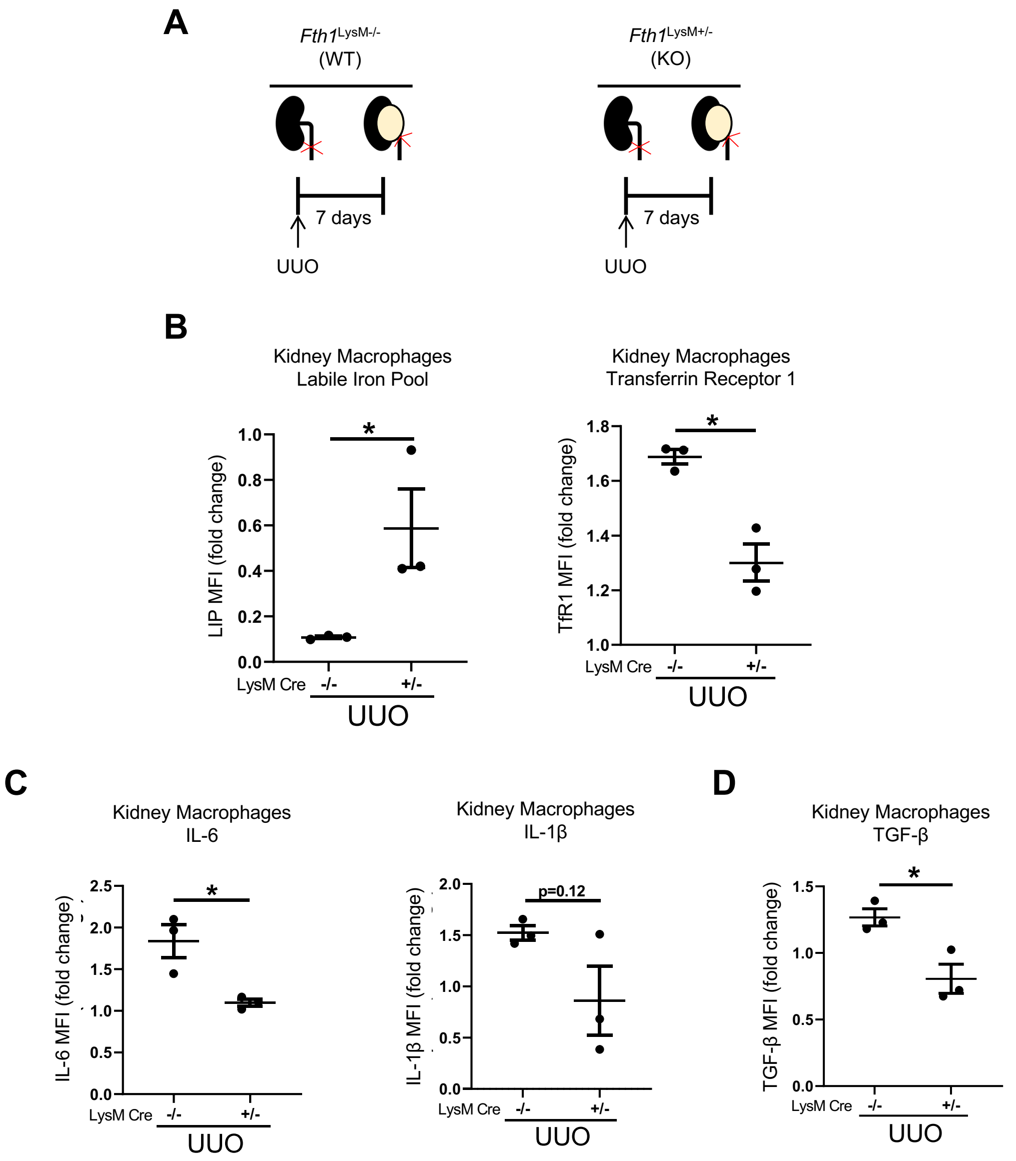

Supplemental Figure 12. Ferritin heavy chain suppression repleted labile iron pool in kidney macrophages and reduced their inflammatory and fibrotic responses in the unilateral ureteral obstruction model. (**A**) Schematic diagram of unilateral ureteral obstruction (UUO) experiments in wild type (WT, *Fth1*$^{LysM-/-}$) and ferritin heavy chain (*Fth1* gene) LysM-Cre specific knockout (*Fth1*$^{LysM+/-}$) mice. Kidneys were harvested 7 days after UUO. (**B**) *Fth1* deletion repleted labile iron pool (LIP) and correspondingly reduced transferrin receptor 1 (TfR1 or CD71) expression in kidney macrophages upon UUO. (**C**) Expression of IL-6 and IL-1β in kidney macrophages upon UUO in *Fth1*$^{LysM-/-}$ and *Fth1*$^{LysM+/-}$ mice. (**D**) TGF-β expression in *Fth1*$^{LysM-/-}$ and *Fth1*$^{LysM+/-}$ kidney macrophages upon UUO. Error bars represent SEM. Data were analyzed using *t* test, n=3 per group; *P<0.05.

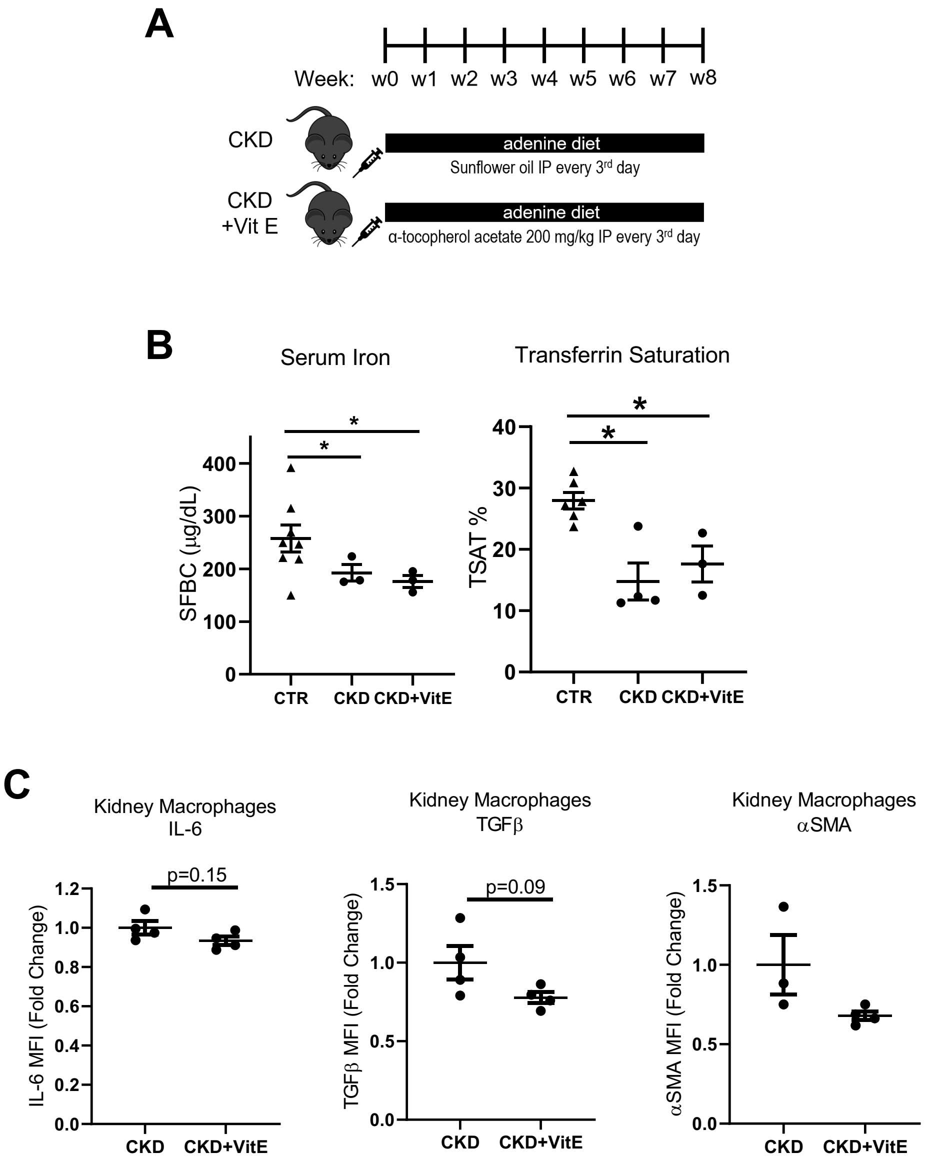

Supplemental Figure 13. Antioxidant treatment with tocopherol acetate improves kidney function, oxidative stress, and inflammation in kidney macrophages in mice with chronic kidney disease, similar to iron dextran therapy. (**A**) Chronic kidney disease (CKD) was induced by the 0.2% adenine diet that mice received for 8 weeks. A subset of the mice received intraperitoneal injections of α-tocopherol (vitamin E) 200 mg/kg every 3rd day throughout the experimental period. Blood for serum separation was collected at euthanasia. Kidneys were processed for digestion immediately upon harvesting and kidney single cell suspensions were analyzed by flow cytometry. (**B**) α-Tocopherol did not affect serum iron and transferrin saturation in CKD mice. (**C**) Expression of IL-6, TGF-β, and α-smooth muscle actin (α-SMA) in kidney macrophages of CKD mice in the presence and absence of α-tocopherol therapy. Data were analyzed by ANOVA (A) and *t* test (B-F); n=3-4 per group. Error bars represent SEM; **P*<0.05.

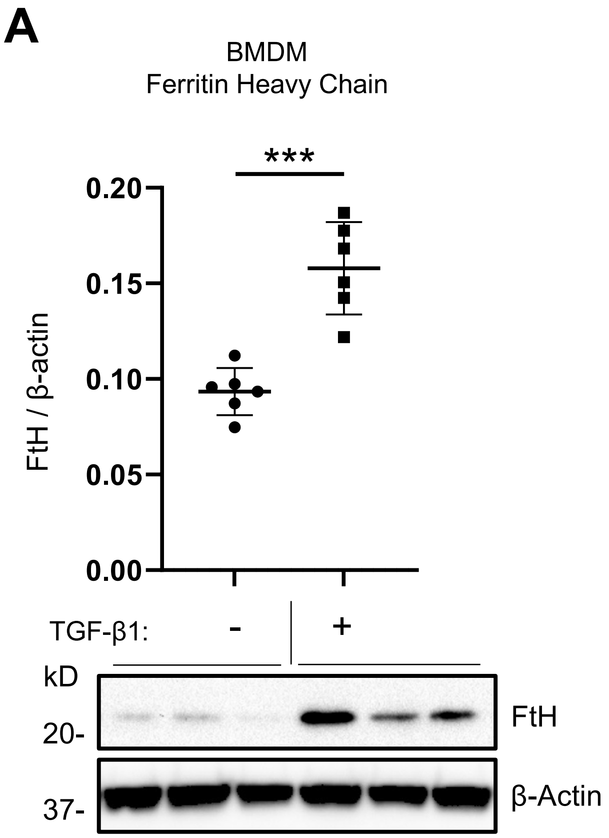

Supplemental Figure 14. TGF-β induces ferritin heavy chain expression while reducing labile iron pool in bone marrow derived macrophages. Bone marrow derived macrophages (BMDMs) were left untreated (TGF-β1-) or exposed to 5 ng/mL of TGF-β1 for 20 hours (TGF-β1+). No iron was added to the media for either group. (**A**) Ferritin heavy chain (FtH) protein expression in two groups of cells assessed by western blot (n=6 per group). (**B**) Flow cytometry analysis of labile iron pool (LIP) in F4/80-positive control BMDM and BMDM exposed TGF-β1; n=4 per group. MFI, mean fluorescent intensity. Data were analyzed using *t* test. Error bars represent SD; **P*<0.05; ****P*<0.001.

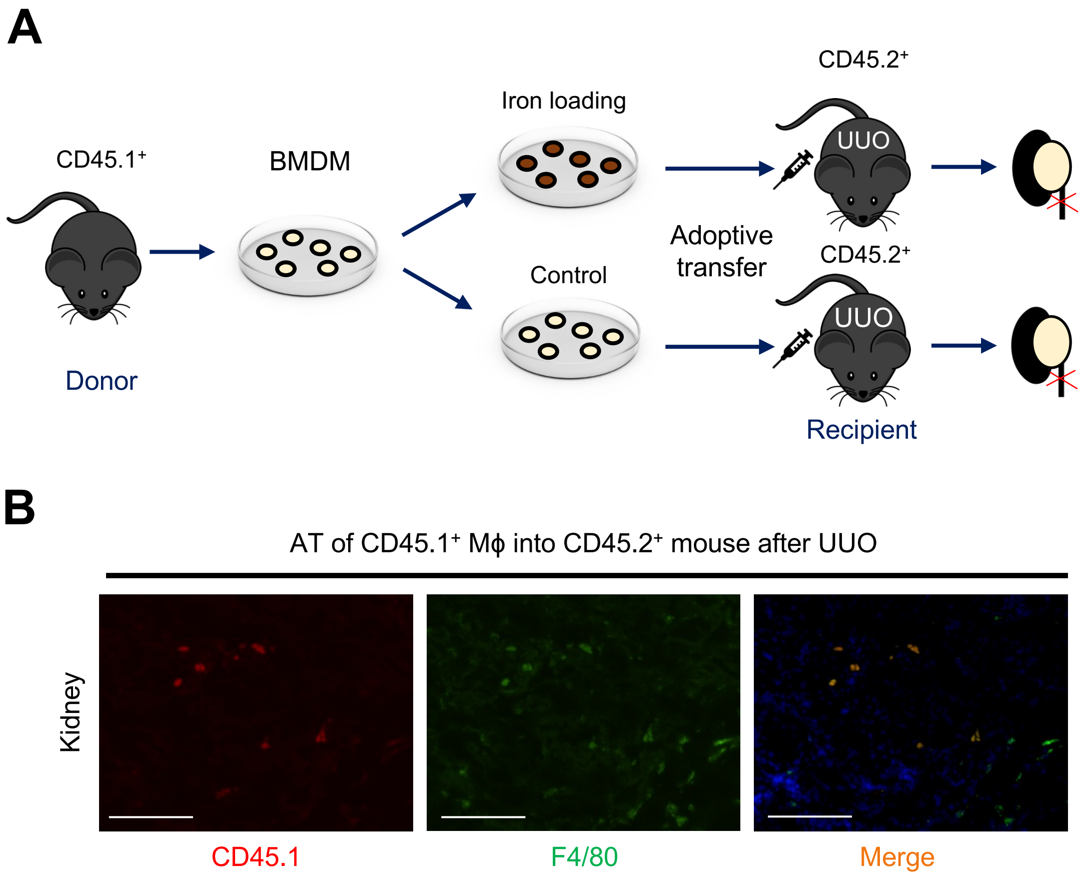

Supplemental Figure 15. Adoptive transfer of bone marrow-derived macrophages leads to macrophage infiltration of kidney tissue following unilateral ureteral obstruction. (**A**) Bone marrow-derived macrophages (BMDM) were isolated from CD45.1$^+$ mice and left untreated (control) or treated with 25μM ferric ammonium citrate (iron-loaded) for 20 hours prior to adoptive transfer. Kidney fibrosis was induced by unilateral ureteral obstruction (UUO) in CD45.2$^+$ mice. CD45.2$^+$ mice received intravenously control or iron-loaded CD45.1$^+$ macrophages. Kidneys were harvested 5 days after adoptive transfer (AT). (**B**) CD45.1$^+$ donor macrophages were visualized in the recipient kidneys by immunofluorescent microscopy showing colocalization of the donor marker CD45.1 (red) and the macrophage marker F4/80 (green). Scale bars, 100 μm. Mϕ, macrophage.
